# Allelic contribution of *Nrxn1α* to autism-relevant behavioral phenotypes in mice

**DOI:** 10.1371/journal.pgen.1010659

**Published:** 2023-02-27

**Authors:** Bing Xu, Yugong Ho, Maria Fasolino, Joanna Medina, William Timothy O’Brien, Janine M. Lamonica, Erin Nugent, Edward S. Brodkin, Marc V. Fuccillo, Maja Bucan, Zhaolan Zhou

**Affiliations:** 1 Department of Genetics, University of Pennsylvania Perelman School of Medicine, Philadelphia, Pennsylvania, United States of America; 2 Autism Spectrum Program of Excellence (ASPE), University of Pennsylvania Perelman School of Medicine, Philadelphia, Pennsylvania, United States of America; 3 Department of Urology, The First Affiliated Hospital of Shandong First Medical University & Shandong Province Qianfoshan Hospital, Shandong Medicine and Health Key Laboratory of Organ Transplantation and Nephrosis, Shandong Institute of Nephrology, Jinan, Shandong, China; 4 Preclinical Models Core, Intellectual and Developmental Disability Research Center (IDDRC) Children’s Hospital of Philadelphia, University of Pennsylvania, Philadelphia, Pennsylvania, United States of America; 5 Department of Psychiatry, University of Pennsylvania Perelman School of Medicine, Philadelphia, Pennsylvania, United States of America; 6 Department of Neuroscience, University of Pennsylvania Perelman School of Medicine, Philadelphia, Pennsylvania, United States of America; Geisel School of Medicine at Dartmouth, UNITED STATES

## Abstract

Copy number variations (CNVs) in the Neurexin 1 (*NRXN1*) gene, which encodes a presynaptic protein involved in neurotransmitter release, are some of the most frequently observed single-gene variants associated with autism spectrum disorder (ASD). To address the functional contribution of *NRXN1* CNVs to behavioral phenotypes relevant to ASD, we carried out systematic behavioral phenotyping of an allelic series of *Nrxn1* mouse models: one carrying promoter and exon 1 deletion abolishing *Nrxn1*α transcription, one carrying exon 9 deletion disrupting *Nrxn1*α protein translation, and one carrying an intronic deletion with no observable effect on *Nrxn1*α expression. We found that homozygous loss of *Nrxn1*α resulted in enhanced aggression in males, reduced affiliative social behaviors in females, and significantly altered circadian activities in both sexes. Heterozygous or homozygous loss of *Nrxn1*α affected the preference for social novelty in male mice, and notably, enhanced repetitive motor skills and motor coordination in both sexes. In contrast, mice bearing an intronic deletion of *Nrxn1* did not display alterations in any of the behaviors assessed. These findings demonstrate the importance of *Nrxn1*α gene dosage in regulating social, circadian, and motor functions, and the variables of sex and genomic positioning of CNVs in the expression of autism-related phenotypes. Importantly, mice with heterozygous loss of *Nrxn1*, as found in numerous autistic individuals, show an elevated propensity to manifest autism-related phenotypes, supporting the use of models with this genomic architecture to study ASD etiology and assess additional genetic variants associated with autism.

## Author summary

Deletions of one of the two copies of the Neurexin1 (*NRXN1)* gene are among the most common genetic anomalies in autism spectrum disorder (ASD), a neurodevelopmental condition afflicting 1 in every 44 children in the United States. How *NRXN1* deletions, which can occur at various positions across the gene, contribute to behaviors associated with autism remains unknown. Here we investigated mouse models carrying different deletions across the *Nrxn1* gene, mimicking three distinct ASD-associated deletions observed in humans, and assessed autism-related mouse behaviors. We found that ASD-relevant behavioral traits in mice are dependent on the sex of the animal and the position of the deletion, and that losing one copy of the *Nrxn1* gene (producing a similar genetic structure to humans with ASD) elicits behaviors akin to the core symptoms of autism: impaired social communication and restricted/repetitive behaviors. Additionally, circadian activity is markedly altered in mice lacking functional *Nrxn1* product, consistent with sleep disturbance being the most common comorbidity in autism. Our findings support the importance of sex, genetic structure, and the amount of functional *NRXN1* product in eliciting ASD-relevant behaviors, and the need for animal models carrying anomalies at multiple genes to elucidate the genetic underpinnings of autism.

## Introduction

Neurexins are presynaptic adhesion molecules that play an essential role in trans-synaptic signaling, cell adhesion, and neurotransmitter release [1–3]. There are three evolutionarily conserved Neurexin genes, *NRXN1*, *NRXN2*, and *NRXN3*, that are expressed in the mammalian brain, each of which is transcribed from two distinct promoters that generate a longer alpha isoform (α) and a shorter beta (β) isoform. These isoforms share identical intracellular domains, but different extracellular structures [2]. The *NRXN1* locus contains an additional third promoter internal to the α and β isoforms that gives rise to a gamma isoform (Neurexin1γ), composed of the same intracellular domains as the other two isoforms, but only having the membrane-proximal sequences of the extracellular domain [4]. Although all three neurexin genes have been associated with psychiatric disorders, *NRXN1* variants account for the majority of disease burden, and *NRXN1* variants appear to be the most frequently observed single-gene variants associated with autism spectrum disorder (ASD) [5,6].

*NRXN1* is one of the longest human genes, with 24 exons spanning over 1 Mb on chromosome 2. This lengthy locus is particularly susceptible to non-recurrent copy number variations (CNVs) [2]. Varying in size and location across the *NRXN1* locus, these non-recurrent CNVs are associated with a variety of psychiatric and neurodevelopmental phenotypes, such as developmental delay, intellectual disability, schizophrenia, and autism [2]. Notably, most of the reported CNVs are heterozygous, with only 11 individuals carrying documented homozygous deletions thus far, all of which show significant development delay and are identified as having Pitt-Hopkins syndrome [2,7]. Additionally, exonic deletions, particularly in the 5’ end of *NRXN1*, appear to be more pathogenic than intronic deletions [6]. This supports the idea that disruption of the *NRXN1* alpha isoform (*NRXN1α*) is more frequently associated with clinical phenotypes than disruptions of other isoforms, as the promoters of the *NRXN1β* and *NRXN1γ* isoforms are located downstream in intron 17 and intron 23 of the alpha isoform, respectively [2,4]. Nevertheless, deletions in the 3’ end of the *NRXN1* gene, including those occurring within the *NRXN1β* isoform, have also been associated with clinical manifestations [6,8]. Together, these findings highlight the genetic complexity at the *NRXN1* locus and support the need to systematically assess the neurobiological effect of CNVs in *NRXN1* that vary in location across the genomic locus– 5’ versus 3’ or exonic versus intronic–in eliciting behavioral phenotypes.

The first *Nrxn1* mouse model, in which the promoter and exon 1 is deleted and thus abolishing *Nrxn1α* transcription, was developed two decades ago [9]. Several behavioral phenotyping studies reported that mice carrying *Nrxn1*α promoter/exon 1 homozygous deletion (often referred to as knockout, KO, and named as ΔExon1/ΔExon1 hereafter) exhibit multiple behavioral phenotypes relevant to autism, including alterations in social affiliative behaviors, aggression, locomotion, self-grooming, motor learning, and value-based decision making [10–13]. Although mice with *Nrxn1*α promoter/exon 1 heterozygous deletion (*ΔExon1/+*) were included in several studies, the phenotypes reported in those studies were inconsistent with each other and sex as a variable was often not considered [11,12,14–16]. Moreover, the effects of genomic position of a CNV deletion across the *NRXN1* locus on isoform expression and subsequent autism-associated behavioral phenotypes remain to be assessed.

Given that sleep and circadian disturbances are some of the most common comorbidities in ASD, occurring in 50–80% of autistic children [17–20], the assessment of circadian abnormalities in autism models is of paramount importance. Common sleep problems in ASD include delayed sleep onset, shorter sleep duration, multiple and prolonged night awakenings, and daytime tiredness [20–22]. A recent meta-analysis of 49 peer-reviewed articles and 51 independent samples found that sleep problems are most strongly associated with externalizing symptoms, internalizing symptoms, and executive functioning in autistic individuals [23]. Thus, identifying a translation model that captures the sleep/circadian phenotypes similar to those observed in ASD may elucidate the biological underpinnings of these phenotypes. However, this clinically relevant phenotype has not been assessed in *Nrxn1*α mouse models.

To dissect the genetic complexity of autism at the *NRXN1* locus, we have collected and generated an allelic series of mouse models carrying distinct CNVs across the *Nrxn1* locus, including ΔExon1, that has been studied previously but not systematically for ΔExon1 heterozygotes, and two new mouse models: the first bearing a deletion of *Nrxn1α* exon 9 that disrupts Neurexin 1α protein translation (named as ΔExon9 hereafter), and the second carrying an ASD proband-associated ~20kb deletion in intron 17 of *Nrxn1α*, upstream of the *Nrxn1*β promoter (named as ΔIntron17 hereafter). Given that heterozygous CNVs in *NRXN1* are the most observed genetic architecture associated with ASD in humans, we performed systematic phenotyping of mice carrying heterozygous ΔExon1, ΔExon9 or ΔIntron17, in comparison to their respective wild-type (WT) littermate controls. We have also included homozygous deletions of exon 9 or intron 17 in the same cohort of study as these two mouse models are newly generated. To address sex as a variable and examine behavioral phenotypes relevant to clinical symptoms of ASD, both male and female mouse models were subjected to the same set of behavioral tests, including elevated zero maze to assess anxiety-related behaviors, open field test to measure locomotor activity, three chamber approach for social choice and social memory, rotarod for motor coordination and motor skill learning, running wheels for circadian activity, and resident intruder test for aggressive types of behaviors. We find that heterozygous loss of *Nrxn1*α, as shown in *ΔExon1/+* and *ΔExon9/+* mice, impairs social memory in male mice and enhances repetitive motor coordination in both sexes. Homozygous loss of *Nrxn1*α, as shown in *ΔExon9/ΔExon9* mice, impairs social memory, alters circadian activities, and enhances motor coordination in both sexes, but selectively elicits enhanced aggression in males and reduced affiliative social interactions in females. In contrast, locomotor function and anxiety-related behaviors were not affected in ΔExon1 and ΔExon9 mouse models, regardless of sex or heterozygous or homozygous deletions. Mice with ΔIntron17 do not show any behavioral abnormalities in these tests. Our findings demonstrate a vital role for Neurexin1α in regulating circadian activities, for the first time, and underscore the significance of *Nrxn1*α gene dosage, genomic positioning of CNV deletions, and sex in eliciting ASD-relevant behavioral phenotypes.

## Results

### *Nrxn1* allelic series in mice–expression of *Nrxn1* isoforms

**Fig 1:**
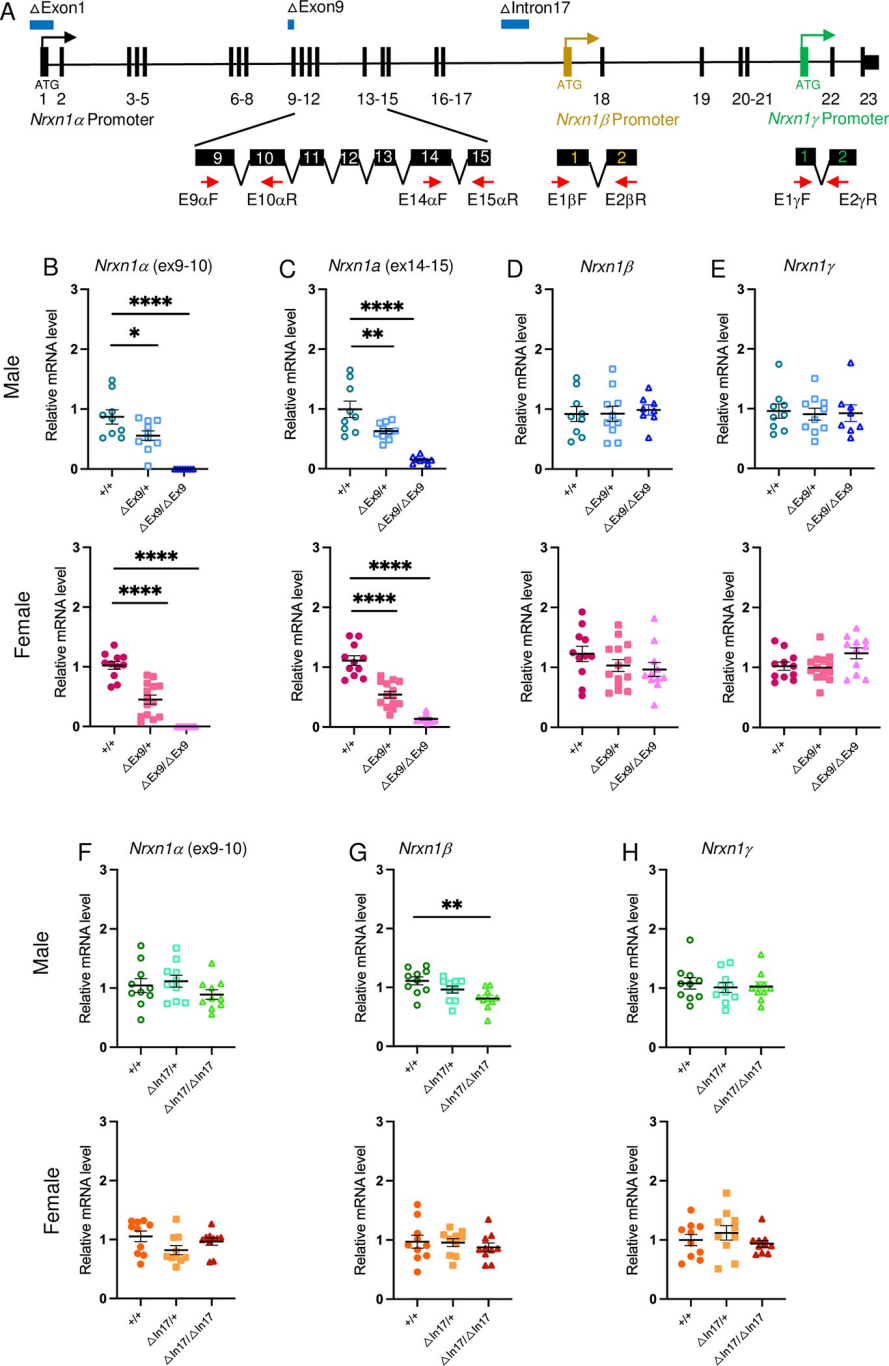
Diagram illustrating *Nrxn1* allelic series and examination of *Nrxn1* isoform specific expression. **A.** Genomic organization of the mouse *Nrxn1* gene. Exons are labeled with vertical lines and numbers. Three different promoters driving *Nrxn1* isoform-specific transcription are indicated with differently colored arrows. The *Nrxn1β* specific exon is labeled with a brown vertical line and the *Nrxn1γ* specific exon is labeled with a green vertical line. Translation start site, ATG, for each isoform-specific protein translation, was labeled under each isoform-specific exon. The positions of primers used for quantitative RT-PCR are indicated below enlarged exons (black bars) with red arrows. The genomic positions for promoter/exon 1 deletion (ΔExon1), exon 9 deletion (ΔExon9), and a ~20kb deletion in intron 17 (ΔIntron17) are marked with blue bars on top of the *Nrxn1* gene. **B-E.** Quantitative measurement of *Nrxn1* isoform specific mRNAs in the ΔExon9 mouse line (top row for males and bottom row for females). (B) *Nrxn1α* mRNAs containing the exon 9 sequence is decreased to about 50% in ΔExon9/+ mice and 100% in ΔExon9/ΔExon9 mice compared to +/+ WT controls (Exon9 labelled as ΔEx9). (C) *Nrxn1α* mRNAs are reduced to about 50% in ΔExon9/+ and nearly 100% in ΔExon9/ΔExon9 mice compared to +/+ WT controls. *Nrxn1β* (D) and *Nrxn1γ* (E) mRNAs are not altered in mice carrying ΔExon9/+ or ΔExon9/ΔExon9 in comparison to +/+ (WT). For panels of ΔExon9 mice, male +/+, n = 9; male ΔExon9/+, n = 10; male ΔExon9/ΔExon9, n = 9; female +/+, n = 11; female ΔExon9/+, n = 14; female ΔExon9/ΔExon9, n = 11. One-way ANOVA test with Dunnett’s multiple comparison test was used to analyze the results. **F-H**. Quantitative measurement of *Nrxn1* isoform specific mRNAs in the ΔIntron17 mouse line (top row for males and bottom row for females). *Nrxn1α* (F), *Nrxn1β* (G) and *Nrxn1γ* (H) mRNAs are not altered in mice carrying ΔIntron17/+ or ΔIntron17/ΔIntron17, in comparison to +/+ (WT) (Intron17 labelled as In17). For panels of ΔIntron17 mice, n = 10 in each genotype group. One-way ANOVA test with Dunnett’s multiple comparison test was used to analyze the results. For all panels in Fig 1, data are represented as mean ± SEM; *p<0.05; **p<0.01; ***p<0.001; ****p<0.0001.

In this study, we employed three mouse models with distinct CNVs across the *Nrxn1* locus to assess the functional significance of genomic positioning of those CNVs. The first was a newly acquired mouse model from the MRC Mary Leon Center carrying the deletion of exon 9 in *Nrxn1α (Nrxn1*^*ΔExon9/+*^ or *Nrxn1*^*ΔExon9/ΔExon9*^; subsequently referred to as *ΔExon9/+* or *ΔExon9/ΔExon9*), an exon that is conserved across humans and mice [24]. Deletion of exon 9 introduces a premature stop codon in exon 10, thus disrupting Neurexin1α protein translation at its N-terminus. To measure the extent to which *Nrxn1* mRNAs, particularly the three different isoforms, are affected by exon 9 deletion, we carried out RT-qPCR to measure the relative *Nrxn1α* isoform-specific mRNA levels in cortical tissues of ΔExon9 mice using primers located in different conserved exons (Fig 1A). Specifically, we used primer pair E9α-F and E10α-R to detect exon 9 deletion, finding that this PCR product was reduced by roughly 50% in heterozygous mice (*ΔExon9/+*) and by nearly 100% in homozygous mice (*ΔExon9/ΔExon9*), regardless of sex (Fig 1B), confirming the successful deletion of exon 9 in this ΔExon9 model.

Notably, RT-PCR with primers located in exon 14 and exon 15 (primer pair E14α-F and E15α-R; Fig 1A), two conserved exons across humans and mice [24], shows that relative *Nrxn1α* mRNA levels are significantly decreased to about 50% of WT levels in *ΔExon9/+* mice and nearly eliminated in *ΔExon9/ΔExon9* mice, in both males and females (Fig 1C). In contrast, the relative mRNA expression for both *Nrxn1β* and *Nrxn1γ* isoforms are not affected regardless of heterozygous or homozygous deletion of exon 9 in both sexes (Fig 1D and 1E). These results suggest that the premature stop codon in exon 10 triggers nonsense-mediated mRNA decay (NMD), a known surveillance mechanism that targets mRNAs containing a premature stop codon for rapid degradation [25,26]. Thus, NMD minimizes potential production of truncated protein products of Neurexin 1α. Unfortunately, the lack of specific antibodies against Neurexin 1α limited our ability to examine Neurexin 1α protein levels in this study. Given the premature stop codon/NMD triggered by deletion of exon 9 and the resulting elimination of *Nrxn1α* mRNAs (Fig 1C), Neurexin1α protein translation is likely abolished and mice with ΔExon9 are therefore considered as a legitimate loss-of-function model to study the function of *Nrxn1α*.

To determine whether a CNV in an intron near the 3’ end of the *Nrxn1* gene elicits behavioral phenotypes, we also generated a mouse model carrying a ~20 kb deletion at intron 17 of *Nrxn1α* (*Nrxn1*^*ΔIntron17/+*^ *or Nrxn1*^*ΔIntron17/ΔIntron17*^; subsequently referred to as *ΔIntron17/+* or *ΔIntron17/ΔIntron17*) that is similar to a CNV identified in an individual on the autism spectrum (in the Autism Spectrum Program of Excellence (ASPE) cohort [27]). Upon examining the expression levels of all three different *Nrxn1* isoforms in this mouse model, we found that both *Nrxn1α* and *Nrxn1γ* transcript levels were unaffected in these mice (Fig 1F and 1H). However, *Nrxn1β* levels were slightly reduced in homozygous male mice (*ΔIntron17/ΔIntron17*), compared to WT mice (Fig 1G). This reduction may be partially related to the fact that the 20kb intronic deletion is approximately 80kb upstream from the promoter of *Nrxn1β*, which is located within intron 17 of *Nrxn1α* [9,28,29]. However, the slight reduction of *Nrxn1β* isoform expression is not observed in female mice carrying the same intronic homozygous or heterozygous deletions (Fig 1G). Nevertheless, this mouse model allows us to probe behavioral phenotypes associated with *Nrxn1* distal intronic deletion found from an ASD proband.

Previously, multiple studies have examined the behavioral phenotypes in mice carrying homozygous *Nrxn1α* promoter/exon 1 deletions [9–16]. However, heterozygous ΔExon1 mice have not been systemically studied in both males and females. Given the large number of CNVs occurring near the 5’-end of *NRXN1* locus in humans with autism and that nearly all of those CNVs are heterozygous, we have included this mouse model, particularly the heterozygotes (*ΔExon1/+*), in our allelic series as well.

### Normal locomotion and anxiety-like behaviors in *Nrxn1* mutant mice

To systematically characterize the contribution of gene dosage and genomic position of CNV deletions in *NRXN1* to autism-relevant behavioral phenotypes in both males and females, we performed a battery of behavioral tests (S1 Fig) in adult mice (≥ 3 months of age) from these three mouse models with WT, heterozygous, and homozygous deletions of specific parts of *Nrxn1*. Given that anxiety is a comorbid condition in a subset of individuals on the autism spectrum, we explored whether the *Nrxn1* mutant mice display alterations in anxiety-related behaviors in the elevated zero maze and spontaneous activity in the open field test. The elevated zero maze is sensitive to anxiolytic treatments, which increase time spent in the open areas of the maze. In the open field, the natural proclivity of a mouse to explore a novel environment is assessed, providing data on ambulation/locomotor function and thigmotaxis. Thigmotaxis refers to the tendency to move along the periphery of the arena and increased thigmotaxis is considered an anxiety-related phenotype. We found that all three mouse models, ΔExon1, ΔExon9, and ΔIntron17, exhibited similar anxiety-like behaviors as WT littermates on the elevated zero maze and open field tests, regardless of sex or genotype (S2 and S3 Figs). Mice carrying heterozygous ΔExon1 show slightly increased locomotor activity in male carriers (S2G Fig), but not in females (S2K Fig), and no locomotion difference was found in mice carrying ΔExon9 or ΔIntron17 (S2 and S3 Figs). This is in stark contrast to other mouse models of neurodevelopmental disorders, such as those for Rett syndrome that show robust reductions in locomotor activity and those for CDKL5 deficiency disorder that exhibit consistent hyperactivity across similar behavioral tasks [30–33]. Together, the absence of significant alterations in locomotor or anxiety-related behaviors in these *Nrxn1* mouse models provides a behavior control and allows us to appropriately interpret other motor-related behavioral phenotypes.

### Impairments of social function in *Nrxn1α* mutant mice

**Fig 2:**
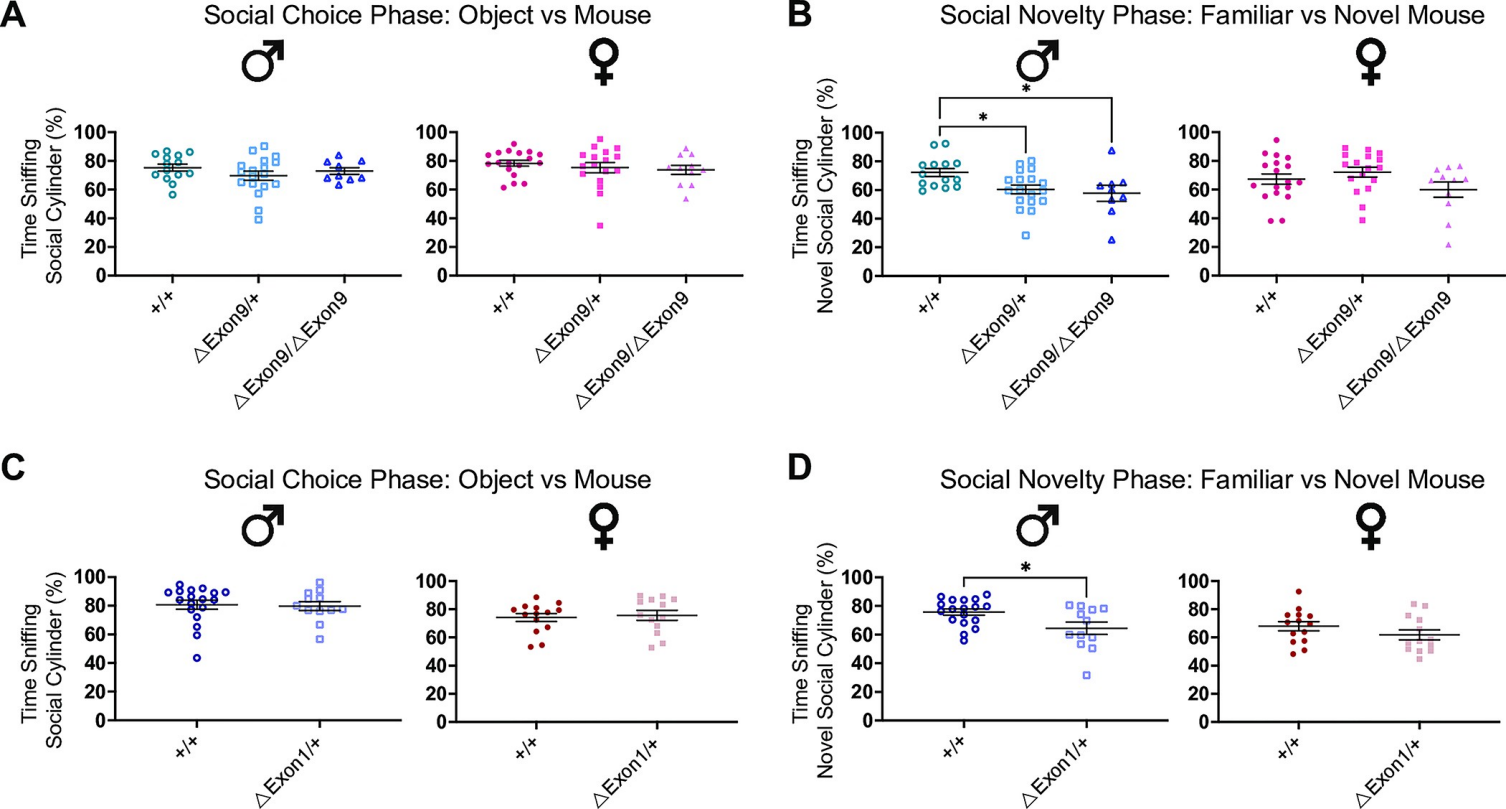
Reduction in *Nrxn1α* expression impairs the preference for social novelty in male mice. **A.** No genotype difference was observed in time spent sniffing social cylinder among ΔExon9 male (left) and female (right) mice in the social choice phase. **B.** ΔExon9/+ and ΔExon9/ΔExon9 male mice spend significantly less time than +/+ males sniffing the novel mouse (left), and no difference was found in females (right) in the social novelty phase. Male +/+, n = 14; male ΔExon9/+, n = 18; male ΔExon9/ΔExon9, n = 9; female +/+, n = 18; female ΔExon9/+, n = 17; female ΔExon9/ΔExon9, n = 11. Data were analyzed using one-way ANOVA test with Tukey’s multiple comparison test. **C.** No genotype difference was observed in time spent sniffing social cylinder among ΔExon1 male (left) and female (right) mice in the social choice phase. **D**. ΔExon1/+ male mice spend significantly less time than +/+ males sniffing the novel mouse (left), and no difference was found in females (right) in the social novelty phase. Male +/+, n = 18; male ΔExon1/+, n = 12; female +/+, n = 14; female ΔExon1/+, n = 13. Data were analyzed using the Mann-Whitney U test. For all panels in Fig 2, the data are represented as mean ± SEM; *p<0.05.

Since persistent deficits in social interactions are key diagnostic criteria for ASD, we next examined social behavior in *Nrxn1* mutant mice using the three-chamber social approach and the resident-intruder test. There are two phases of the social approach test in which behaviors are assessed: (1) a social choice phase that is indicative of social interaction in which the animal explores cylinders containing either a novel, inanimate object or novel conspecific mouse, and (2) a social novelty phase in which the animal has the choice to explore cylinders containing either a familiar, conspecific mouse or a novel, conspecific mouse [34,35]. None of the mouse models exhibited impairments in the social choice phase of the three-chamber social approach paradigm, showing comparable time spent sniffing the social cylinder across different genotypes, regardless of *Nrxn1α* expression status, and indicating normal olfactory function in discriminating a social cue as well (Figs 2A, 2C and S4; S1–S3 Tables). On the other hand, mice with reduced *Nrxn1α* expression, such as those carrying heterozygous or homozygous ΔExon9 or heterozygous ΔExon1, displayed a reduction in time spent exploring the novel mouse during the social novelty phase, particularly in male carriers, indicative of an impairment in preference for social novelty or social memory [34,35] (Fig 2B and 2D; S1 and S2 Tables). In contrast, male and female mice carrying intronic deletions do not show any deficits in these three chamber social tests (S4 Fig; S3 Table).

**Fig 3:**
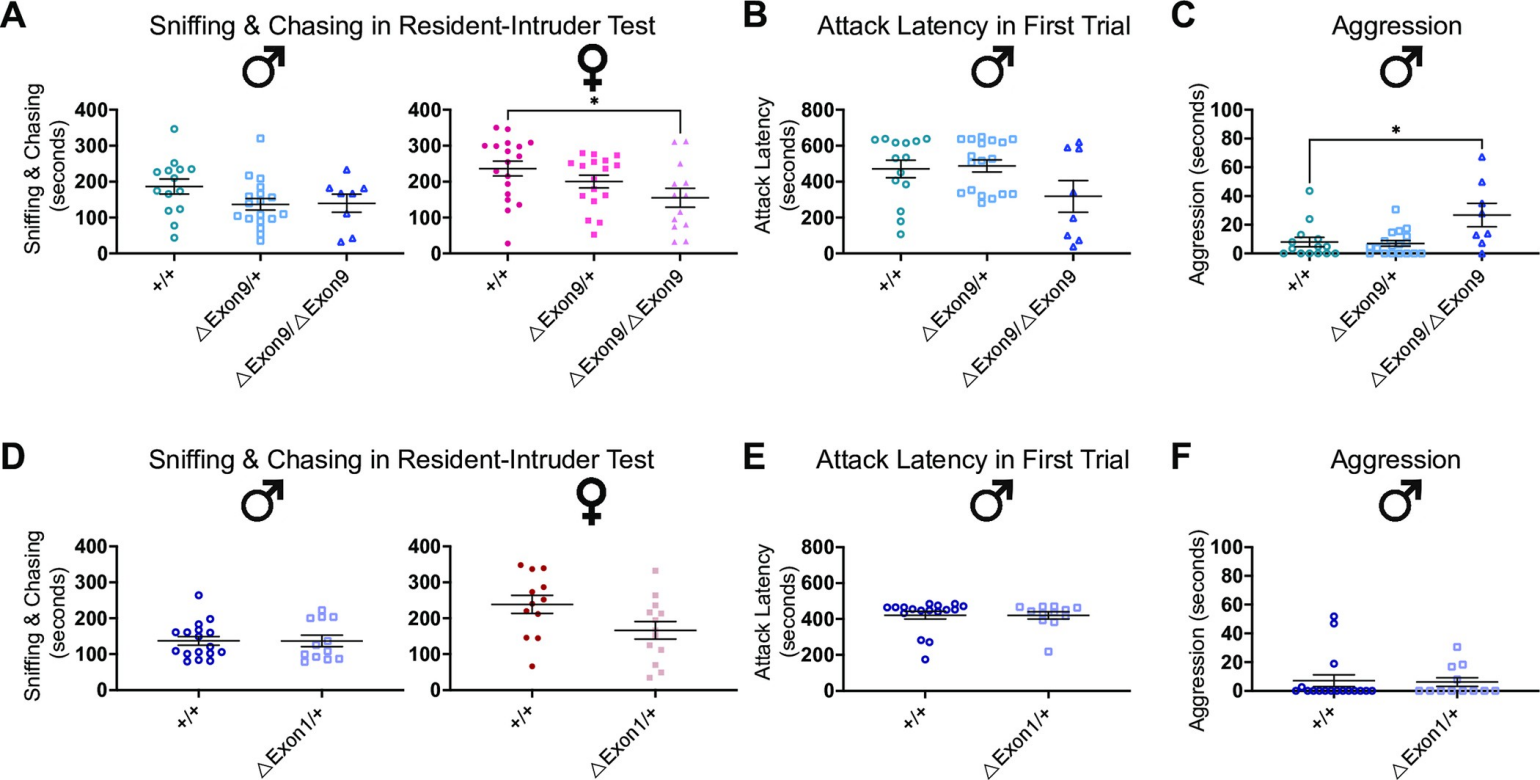
Complete loss of *Nrxn1α* expression enhances aggressive behaviors in males and reduces passive interactive behaviors in females. **A-C.** No genotype difference was observed in time spent sniffing and chasing the intruder mouse (A, left) or for attack latency (B) among ΔExon9 males. ΔExon9/ΔExon9 female mice show reduced time sniffing and chasing the intruder mouse (A, right). ΔExon9/ΔExon9 male mice exhibit increased aggressive behaviors compared to +/+ males (C). Male +/+, n = 14; male ΔExon9/+, n = 18; male ΔExon9/ΔExon9, n = 8; female +/+, n = 18; female ΔExon9/+, n = 17; female ΔExon9/ΔExon9, n = 13. The data were analyzed using one-way ANOVA test with Tukey’s multiple comparison test. **D-F.** No genotype difference was observed in time spent sniffing and chasing the intruder mouse among ΔExon1 males (left) and females (right) (D), for attack latency (E) or for exhibiting aggressive behaviors among ΔExon1 males (F). The number of animals used for each group in this test is the same as those in Fig 2C and 2D. Data were analyzed using Mann-Whitney U test. For all panels in Fig 3, data are represented as mean ± SEM; *p<0.05.

The resident-intruder assay is a test in which a sex-matched young intruder mouse is introduced into a singly housed mouse’s home cage to assess their interactive behaviors, including aggressive interactions, such as barbering, and passive interactions, such as chasing, following, and anogenital sniffing. Neither *ΔExon9/+*, *ΔExon1/+*, nor *ΔIntron17/+*, *ΔIntron17/ΔIntron17* mice displayed alterations in aggressive behaviors and passive interactions in the resident-intruder test (Figs 3 and S5; S2 and S3 Tables). However, mice with *ΔExon9/ΔExon9* exhibited sex-specific alterations with males demonstrating increased aggressive behaviors and females demonstrating reduced passive social interactions (Fig 3A–3C). Furthermore, in the ΔExon9 mouse strain there was a significant effect of genotype on time the resident mouse spent with the intruder mouse (S1 Table). Together, these findings suggest that *Nrxn1α* dosage plays a critical role in altering particular social behaviors. For example, decreased preference for social novelty is observed in mice with reduced *Nrxn1α* expression, including heterozygous deletion of exon 1 or exon 9, as well as homozygous deletion of exon 9 (*ΔExon1/+*; *ΔExon9/+*, *ΔExon9/ΔExon9*). However, the manifestation of aggressive behaviors requires a homozygous loss of *Nrxn1α*. These observations support that a dose-dependent reduction in *Nrxn1α* expression leads to multiple impairments in social function that are relevant to autism.

### A reduction in *Nrxn1α* expression alters circadian locomotor and bout activity

**Fig 4:**
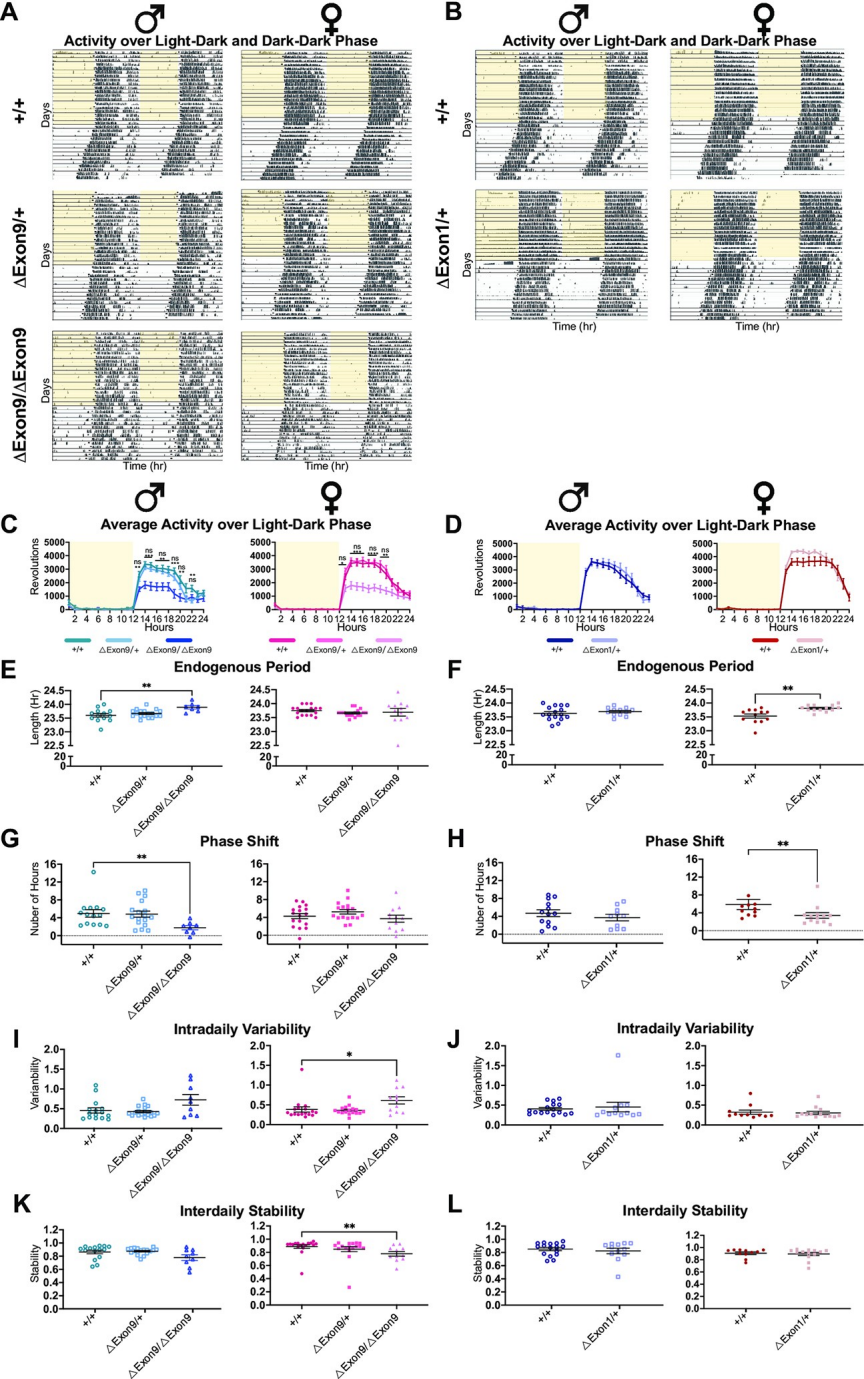
A reduction in *Nrxn1α* expression alters circadian locomotor activity. **A-B.** Representative actograms from mice under two different lighting conditions: (1) 12 h of light (indicated by the yellow shading) followed by 12 h of dark (L/D) and (2) constant dark (D/D). Actograms depict locomotor activity (wheel revolutions) across time with days stacked vertically and double plotted with the x axis spanning 2 days (48 hours). Grey squares indicate activity onsets. **C-D.** Activity profiles averaged over 5 consecutive days of L/D conditions across sex and genotype. Repeated measures two-way ANOVA test with Tukey’s multiple comparisons test (C) or Sidak’s multiple comparison test (D). *p<0.05; **p<0.01; ***p<0.001; ****p<0.0001. **E-F.** Endogenous period across sex and genotype. (E) Mean endogenous periods per group: male +/+, 23.60; male ΔExon9/+, 23.67; male ΔExon9/ΔExon9, 23.89; female +/+, 23.75; female ΔExon9/+, 23.66; female ΔExon9/ΔExon9, 23.69. Kruskal-Wallis test with Dunn’s multiple comparison test; **p<0.01. (F) Mean endogenous periods per group: male +/+, 23.63; male ΔExon1/+, 23.69; female +/+, 23.53; and female ΔExon1/+, 23.81. Welch’s t test; **p<0.01. **G-H.** Phase shift across sex and genotype. (G) Kruskal-Wallis test with Dunn’s multiple comparison test; **p<0.01. (H) Mann-Whitney U test; **p<0.01. **I-J.** Intradaily variability in the L/D phase across sex and genotype. (I) Kruskal-Wallis test with Dunn’s multiple comparison test; *p<0.05. (J) Mann-Whitney U test. **K-L.** Interdaily stability in the L/D phase across sex and genotype. (K) Kruskal-Wallis test with Dunn’s multiple comparison test; **p<0.01. (L) Mann-Whitney U test. The number of animals used for each group in this test is the same as those in Fig 2. For all panels in Fig 4, data are represented as mean ± SEM.

Sleep disturbances are one of the most common comorbidities in autism and estimated to occur in up to 80% of autistic children [17–20]. Reported sleep disturbances in autism include perturbed sleep-onset, impaired sleep maintenance, and/or alterations in waking time [17–20]. Furthermore, disturbances in circadian rhythms have recently been reported in a mouse model of neurodevelopmental disorders induced via prenatal maternal immune activation [36]. As this clinically relevant phenotype has not been previously studied in *Nrxn1* deficient animal models, we sought to systemically characterize endogenous circadian activity cycles and assessed multiple parameters, including both parametric and nonparametric traits of circadian activity using running wheel cages in which mice are individually housed and wheel revolutions are continually collected over 4 weeks of time (S1 Fig). In the first two weeks, mice were exposed daily to 12 hours of light and 12 hours of dark (L/D phase), which mimics the naturally occurring light-dark signal from the environment. In the last two weeks, mice were exposed to constant darkness (D/D phase), which enables the assessment of circadian behavior (or the free-running rhythm) without the influence of light entrainment (Figs 4A and 4B; S1, S9A and S9B).

During the L/D phase, we found that only mice with homozygous loss of *Nrxn1α* demonstrated significantly decreased running wheel activity, with *ΔExon9/ΔExon9* mice exhibiting a reduction specifically during the dark period, which is the active period of nocturnal mice (Figs 4Cs, 4D and S9C). Notably, reduced overall activity was also observed in the most active 10 hours of the day (so called M10 [37–39]), but not the least active 5 hours of the day (so called L5 [37–39]), in both the L/D and D/D phases in homozygous ΔExon9 mice (S6A and S6B Fig), but not in mice of other genotypes except L5 in ΔExon1 (S6D and S6E; S7A and S7B, S7G and S7H; S9I and S9J, S9M and S9N Figs). However, the start time of the most active 10 hours of the day and the least active 5 hours of the day, were not significantly altered in any of the genotypes (S7C–S7F, S7I–S7L; S9K and S9L, S9O and S9P Figs).

Assessment of the endogenous free-running circadian period reveals that reduced *Nrxn1α* gene expression lengthens the endogenous period in a sex-dependent manner, as alterations were observed in male *ΔExon9/ΔExon9* mice and female *ΔExon1/+* mice (Figs 4E and 4F and S9D; endogenous periods are reported in figure legends; S1–S3 Tables) Relatedly, examination of the phase shift of activity onsets between the L/D and D/D periods led to the observation that lower *Nrxn1α* gene expression is associated with a reduction in phase shift (a phase delay), meaning the onset of activity across the D/D phase is significantly shifted later in time compared to the onset of activity across the L/D phase; a sex-specific effect was observed in phase shift as well (Figs 4G and 4H, S9E; S1–S3 Tables).

We next measured intradaily variability, a non-parametric measurement of rest-activity rhythm fragmentation that assesses the extent and frequency of transitions between rest and activity within a 24-hour period. High intradaily variability is indicative of an increase in activity episodes during typically restful periods like sleep, and/or an increase in rest episodes during typically active periods [37–39]. This type of high intradaily variability has been reported in age-related conditions, such as Alzheimer’s disease [37,40,41], and in individuals associated with reduced social interaction, poor cognitive and motor performance [42,43]. We found that only mice with homozygous loss of *Nrxn1α* (*ΔExon9/ΔExon9*) exhibited increased intradaily variability during the L/D phase and/or D/D phase, particularly in females (Figs 4I and 4J; S6C, S6F; S9F and S9G; S1–S3 Tables). We also examined interdaily stability, a non-parametric measurement of how similar rest-activity patterns are from one day to the next, of rest-activity synchronization to the 24-hour light-dark cycle. This synchronization occurs through external inputs–somatosensory, social effects, and physical activity–to the main circadian oscillator, the suprachiasmatic nucleus [37–39]. A reduction in interdaily stability has been observed in aging, cognitive disorders, and dementia [40,44,45]. We observed that female mice with homozygous loss of *Nrxn1α* exhibited reduced interdaily stability, in contrast to heterozygous and WT mice (Figs 4K and 4L; S9H; interdaily stability can only be measured in the L/D phase; S1–S3 Tables).

**Fig 5:**
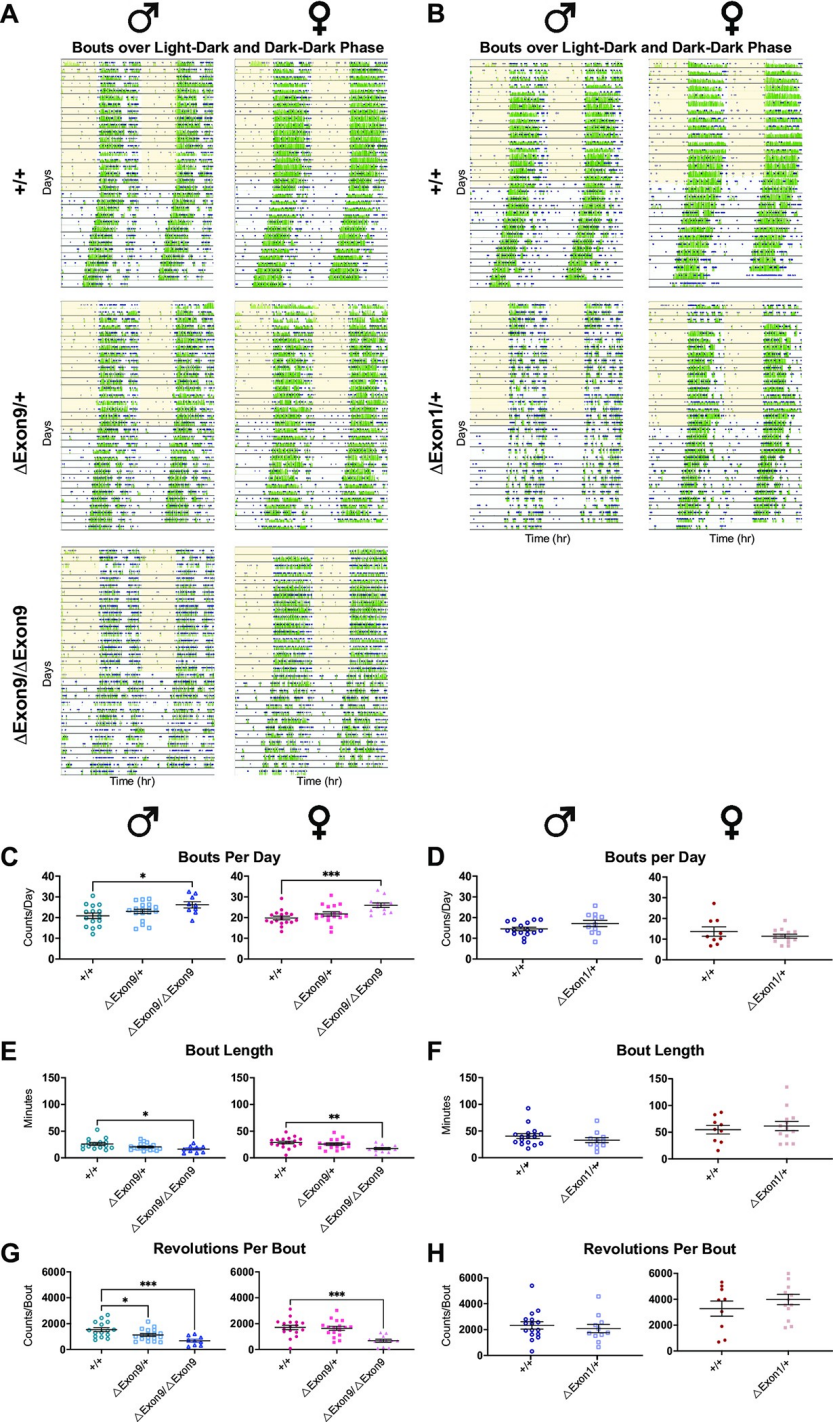
A reduction in *Nrxn1α* expression alters circadian bout activity. **A-B.** Representative actograms with activity indicated in green and bouts marked with blue squares from mice under two different lighting conditions: (1) 12 h of light (indicated by the yellow shading) followed by 12 h of dark (L/D) and (2) constant dark (D/D). Actograms depict locomotor activity (wheel revolutions) in green across time with days stacked vertically and double plotted with the x axis spanning 2 days (48 hours). **C-D.** Bouts per day in the L/D phase across sex and genotype. (C) Ordinary one-way ANOVA with Holm-Sidak’s multiple comparison test; *p<0.05 and ***p<0.001. (D) If groups were normally distributed (D’Agostino & Pearson test) and didn’t have significantly different variance (F test), the unpaired t test was used. If samples were not equally distributed or had different variances, Welch’s t test was used. **E-F.** Bout length in the L/D phase across sex and genotype. (E) Kruskal-Wallis test with Dunn’s multiple comparison test; *p<0.05; one-way ANOVA with Holm-Sidak’s multiple comparison test, **p<0.01. (F) If groups were normally distributed (D’Agostino & Pearson test) and didn’t have significantly different variance (F test), the unpaired t test was used. If samples were not equally distributed or had different variances, Welch’s t test was used. **G-H.** Revolutions per bout in the L/D phase across sex and genotype. (G) One-way ANOVA with Holm-Sidak’s multiple comparison test, *p<0.05 and ***p<0.001. (H) If groups were normally distributed (D’Agostino & Pearson test) and didn’t have significantly different variance (F test), the unpaired T test was used. If samples were not equally distributed or had different variances, Welch’s T test was used. The number of animals used for each group in this test is the same as those in Fig 2. Data are represented as mean ± SEM in all graphs.

Given the observed increase in intradaily variability, we next examined bouts of activity (Figs 5A and 5B; S10A and S10B) and found that both male and female mice with homozygous loss of *Nrxn1α* (*ΔExon9/ΔExon9*) showed an increase in the number of daily bouts, but bouts were shorter in duration with fewer revolutions per bout, particularly in the L/D phase (Fig 5C, 5E and 5G; S1–S3 Tables). Similar alterations in bouts of activity were also found in the D/D phase for *ΔExon9/ΔExon9* mice, but they varied depending on sex, with only female homozygotes showing the same phenotype in both L/D and D/D phases (Figs 5C, 5E and 5G; S8A–S8C; S1–S3 Tables). Notably, heterozygous ΔExon1 male mice displayed an increase in the number of daily bouts with less activity per bout in only the D/D phase (Figs 5D, 5F and 5H; S8D–S8F). In contrast, mice with ΔIntron17 did not show any circadian phenotypes (S9 and S10 Figs). Taken together, we found that mice with complete loss *Nrxn1α* expression display robust alterations in circadian activity, a clinical comorbidity that is commonly observed in autism, as shown in multiple measurements. Mice with partial loss of *Nrxn1α* expression in *Exon9/+* and *Exon1/+* show statistically significant changes in a few measurements depending on sex. These findings underscore key roles for *Nrxn1α* gene dosage and sex in the manifestation and extent of circadian activity alterations.

### Reduced *Nrxn1α* expression enhances performance on the rotarod

**Fig 6:**
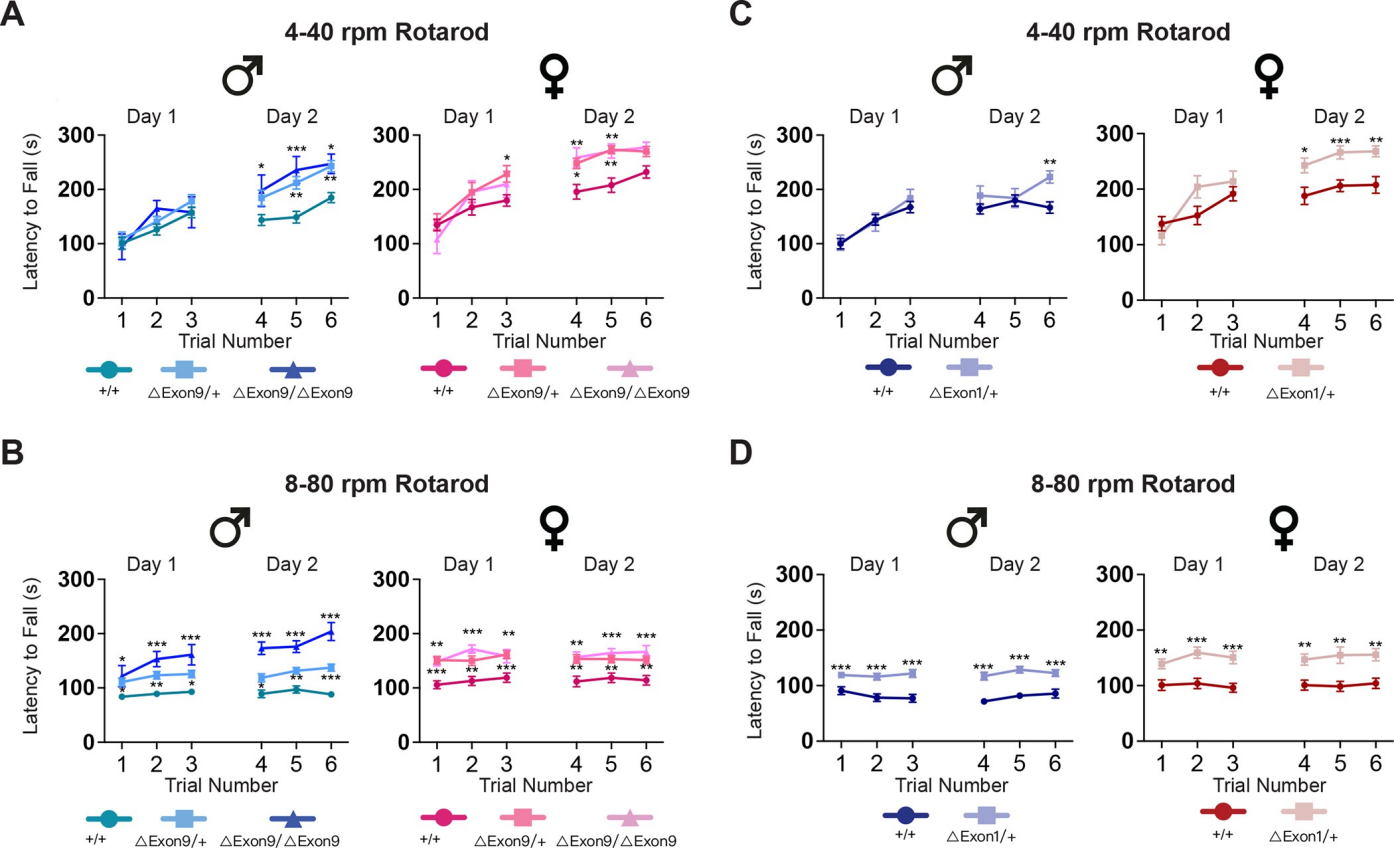
A reduction in *Nrxn1α* expression enhances the motor skill learning and motor coordination. **A-B**. (A) Latency to fall (in seconds) for +/+, ΔExon9/+, and ΔExon9/ΔExon9 male (left) and female (right) mice on the standard accelerating rotarod (4–40 rpm in 5 min). Mice were tested over 3 trials per day for 2 consecutive days. (B) Latency to fall of the same male (left) and female (right) mice from (A) on a higher accelerating rotarod (8–80 rpm in 5 min). Mice were tested over 3 trials per day for the next 2 consecutive days. Two-way repeat measure ANOVA test with Dunnett’s multiple comparison test, *p<0.05; **p<0.01; ***p<0.001. **C-D.** (C) Latency to fall (in seconds) for +/+ and ΔExon1/+ male (left) and female (right) mice on the standard accelerating rotarod (4–40 rpm in 5 min). Mice were tested over 3 trials per day for 2 consecutive days. (D) Latency to fall of the same +/+ and ΔExon1/+ male (left) and female (right) mice from (C) on a higher accelerating rotarod (8–80 rpm in 5 min). Two-way repeat measure ANOVA test with Sidak’s multiple-comparison test, *p<0.05; **p<0.01; ***p<0.001. The number of animals used in each group in the high speed rotarod is the same as those in Fig 2. Data are represented as mean ± SEM in all graphs.

Given that restricted, repetitive patterns of behaviors are key diagnostic criterion in autism, we assessed the extent to which these allelic series of *Nrxn1* mutant mice display alterations on the accelerated rotarod test, a motor coordination and motor skill learning task that serves as a proxy for acquired repetitive behaviors [46]. Previous studies have demonstrated that homozygous deletion of the promoter and exon 1 of Nrxn1α (*ΔExon1/ΔExon1*) display enhanced performance on the rotarod task [10]. Expanding upon this previous finding, we found that heterozygous ΔExon1 mice (*ΔExon1/+*) also exhibited enhanced rotarod performance: compared to control mice, *ΔExon1/+* mice demonstrated similar performance during the initial trials (trials 1–3), but improved performance on subsequent trials in which acceleration stays the same (trials 4–6), implicating enhanced motor skill learning in these mice (Fig 6C). Notably, with increased acceleration on the high speed rotarod following two days on the rotarod with regular speed, *ΔExon1/+* mice show persistently decreased latency to fall across multiple trails over two days, suggesting improved motor coordination in these heterozygous ΔExon1 mice (Fig 6D). Similarly, mice with either heterozygous or homozygous deletion of exon 9 of *Nrxn1α* (*ΔExon9/+* and *ΔExon9/ΔExon9*) also exhibit enhanced motor skill learning and motor coordination (Fig 6A and 6B). In contrast, mice with either heterozygous or homozygous deletion in intron 17 of *Nrxn1α* do not display alterations in rotarod test (S11 Fig). Taken together, our findings suggest that the formation of repetitive motor patterns are sensitive to *Nrxn1α* gene dosage–with approximately 50% reduction of *Nrxn1α* via heterozygous deletion of either exon 9 or promoter/exon 1 leading to this phenotype, regardless of testing in males or females.

## Discussion

Non-recurrent CNV deletions in *NRXN1* are among the most common genetic variants in ASD, underscoring their clinical relevance. However, the extent to which specific variants, differing in their positions across the *NRXN1* locus, contribute to behavioral traits associated with autism remains unknown. This study was designed to investigate the effect of three CNV deletions that vary in location across the *NRXN1* locus on behavior phenotypes relevant to autism in both male and female mouse models. We found that CNV deletions affecting *Nrxn1α* expression, such as heterozygous ΔExon1, heterozygous or homozygous ΔExon9, but not ΔIntron17, were associated with autism-relevant behavioral phenotypes. This could potentially be related to the fact that beta isoforms are not conserved across vertebrates, in contrast to alpha isoforms which exhibit identical intronic architecture in mice and humans [2,47]. These findings align with current genetic studies reporting the majority of CNV deletions in *NRXN1* identified in autism clinical cases occurs near the 5’ end of the *NRXN1*, thus likely affecting expression of the NRXN1*α* isoform [2,4,6,8]. Consistently, we found that deletion of exon 9, an exon conserved in human and mice, results in a premature stop codon and triggers NMD, thus leading to disruption of *Nrxn1α* expression as well.

Given that in humans, homozygous deletions are rare and *NRXN1* heterozygous deletions are associated with ASD clinical phenotypes, behavioral phenotypes observed in heterozygous mice are particularly relevant to translational studies. In this direction, we identified two reproducible behavioral phenotypes in heterozygous mice across two independent mouse models, *ΔExon1/+* and *ΔExon9/+*. In both heterozygous mice, *Nrxn1*α mRNAs were reduced to about 50%. One behavioral alteration found in this study was an impairment in social interactions, with males in both mouse models exhibiting impairments in social novelty seeking or social memory, resolving an inconsistent phenotype from previous studies [12]. The other behavioral alteration observed in this study was enhanced motor coordination, a proxy for acquired repetitive behaviors [46]. We found that this was the most robust phenotype, consistent across two mouse models and present in both males and females, particularly by the high speed rotarod test. Notably, enhanced performance on the high speed rotarod was previously reported in a cohort of mixed male and female mice on mixed Sv129/C57BL/6 genetic background with homozygous ΔExon1. This phenotype is consistently observed in our ΔExon1 and ΔExon9 mice on C57BL/6 background in both sexes. Importantly, these two behaviors–altered social novelty preference and repetitive motor behaviors–are relevant to two core autism diagnostic domains–(1) impairment in social interaction and (2) restricted, repetitive patterns of behaviors. Thus, mice with heterozygous ΔExon1 or ΔExon9 can be valuable models for translational studies of core symptoms in autism.

Our study also documented sex-dependent behavioral traits associated with *Nrxn1α* deficiency in known sexually dimorphic behaviors, as homozygous ΔExon9 males exhibited enhanced aggression but females displayed reduced affiliative social behaviors. This is in line with sex-dependent behavioral responses that have been reported previously in the *Nrxn1α* ΔExon1 deletion models [11,12,14–16]. Growing evidence suggests ASD may be under-identified and under-diagnosed in females [48], with three to four times more males being diagnosed with autism than females [49]. Our findings show that several autism-relevant behaviors differ between males and females. This agrees with prior work that has reported appreciable sex-specific clinical behaviors [50], such as the male bias towards enhanced restricted, repetitive behavior on the autism spectrum [51–55]. The molecular and cellular mechanisms underlying sex-specific differences in autism-relevant behaviors remains unknown, though several models have been proposed to date [55–60]. One working model relevant to our findings comes from a study by Werling and colleagues in which sexually dimorphic gene expression was assessed in neurotypical and autistic human neocortical tissues throughout development. It was found that although ASD risk genes did not display sex-differential expression, genes with sexually dimorphic expression patterns display enhanced dimorphic expression in autistic human neocortical tissue [56], suggesting that sexually dimorphic processes, circuits, and behaviors might be susceptible to autism risk variants. Our study emphasizes the importance of focusing on sex-specific behavioral phenotypes when conducting biomedical and translational studies of *Nrxn1* deficiency, as well as when carrying out clinical diagnosis and measurement of treatment outcomes.

Notably, the two behavioral phenotypes observed in mice with heterozygous loss of *Nrxn1α* were more pronounced in mice carrying homozygous loss of *Nrxn1α*, suggesting a *Nrxn1α* gene dosage-dependent effect on these behaviors. A few other autism-related behaviors, however, were only observed with homozygous loss of *Nrxn1α*, such as increased aggression in males, reduced affiliative social behaviors in females, and altered circadian locomotor and bout activities in both sexes. These findings raise a high likelihood that additional genetic variants in the same or different pathways as *NRXN1* may be contributing to human phenotypes. This is particularly evident in the case of an ASD individual carrying a ~20kb deletion in the intron 17 of *NRXN1*, as mice bearing heterozygous or homozygous deletion of similar genomic region have yet to exhibit any behavioral abnormality. Though this could be related to the genomic architecture and fundamental differences between human and mice, our findings support an oligogenic model where multiple gene variants, heterozygous in nature, work together and enhance susceptibility to autism. Given that Neurexin 1*α* is a presynaptic adhesion molecule that plays a critical role in synapse formation, maintenance, and plasticity, variants in *NRXN1* could lead to alterations in synaptic pathways, and the presence of additional genetic variants within individuals carrying *NRXN1* CNVs could amplify synaptic aberration, resulting in significant perturbations of neurotransmitter release, Ca2+ signaling, glutamate receptor composition, CASK signaling, or excitatory synaptic strength, as impairments in these pathways have been reported in human induced pluripotent stem cell (iPSC) models of *NRXN1* CNV, mouse models of *Nrxn1* [61–67], and other CNVs associated with neuropsychiatric disorders such as 1q21.1 [68]. At present, two functional groups of genes, encoding synaptic proteins or epigenetic factors, are genetically linked to autism. One interpretation we previously reported was that synaptic genes, such as *NRXN1*, harbor broad enhancer-like chromatin domains (BELD) for the facilitation of high level and persistent transcription over time, thus mutations in chromatin genes likely disrupt the BELD feature and indirectly impair synaptic gene function [69]. Through two or more genetic hits, though each affects a single allele encoding synaptic function or chromatin regulation, the combination of both results in significant impairment at the level of synapses, and ultimately elicits autism [69].

In summary, through behavioral phenotypic analysis of an allelic series of *Nrxn1*α mouse models, we show that heterozygous loss of *Nrxn1*α elicits autism-relevant behavioral phenotypes in two core autism diagnostic domains. Various sleep traits were also found to be altered by a reduction in *Nrxn1*α expression, consistent with sleep disturbance comorbidity in autism. These findings support the face validity and the translational utility of *Nrxn1α* haploinsufficiency mouse models, which is important given that heterozygous *NRXN1* variants are the most frequently observed single-gene variants associated with autism. Future work is needed to determine the molecular pathways by which *Nrxn1α* interacts with other gene variants to shape autism-relevant behaviors, the developmental trajectory of those autism-relevant behaviors, and the reversibility of autism-related behavioral phenotypes, at least in animal models. Such future studies may reveal new avenues for mechanism-based therapeutic development to improve the overall quality of life for autistic individuals.

## Materials and methods

### Ethics statement

Animal procedures were conducted in accordance with the ethical guidelines of the National Institute of Health (NIH) and approved by the Institutional Animal Care and Use Committee (IACUC) of the University of Pennsylvania. This study did not include research with human subjects.

### Animals

Mice were group housed in cages of 2 to 5 on a 12-hour light/12-hour dark cycle with food and water provided ad libitum. The mice used in this study for behavioral testing were between 3–5 months of age, including both males and females.

Mice with *Nrxn1α* promoter and exon 1 deletion (ΔExon1) were described previously [9] and have been maintained in *C57BL/6J* background. Mice with *Nrxn1α* exon 9 deletion (ΔExon9) were generated by crossing an exon 9 floxed allele of *Nrxn1α (Nrxn1*^*tm1a(KOMP)Wtsi*^ from MRC Mary Lyon Center, Harwell, UK) [13] with mice carrying *UBC-CreERT2* [70]. An unexpected leaky activity of Cre in male gametes [33] carrying both floxed exon 9 of *Nrxn1α* and *UBC-Cre-ERT2* leads to a germline loss of exon 9 (ΔExon9). The deletion of exon 9 was confirmed by PCR analysis using primers flanking the deleted region and within the exon 9 sequence. To study a CNV identified in an individual on the autism spectrum [27], mouse homologue of the ~20 kb deleted region at intron 17 of *Nrxn1α* was identified and deleted using the CRISPR/Cas9-medicated genomic editing approach. Two sgRNAs (5’ AATATGTGGGCAAGCTGGGT **TGG** 3’ and 5’ GAAATGGTACCTTTGATCTA **AGG** 3’) flanking the deletion region in intron 17 of *Nrxn1α* were injected together with Cas9 protein into 1-cell zygote of *C57BL/6J*/*SJL* genetic background. The target deletion was confirmed by PCR and sequencing analyses using primers flanking the deleted region and deletion carriers (ΔIntron17) were back crossed to *C57BL/6J* for 5 more generations to collect littermates for behavioral phenotyping.

To generate experimental animals used in this study, heterozygous males were bred with heterozygous females to generate mice with homozygous (ΔExon9/ΔExon9; ΔIntron17/ΔIntron17) or heterozygous (ΔExon9/+; ΔIntron17/+) deletions, as well as WT littermates (+/+). We noted that mice carrying homozygous deletion of exon 9 (ΔExon9/ΔExon9) were significantly underrepresented with WT:Het:Homo ratio as 48:90:29, in contrast to the expected ratio of 42:83:42, indicating sub-viability in mice carrying a complete loss of *Nrxn1α*. To generate mice carrying ΔExon1, heterozygous carriers of ΔExon1 were bred with WT mice to collect heterozygotes and WT for experiments described in this study.

### Behavioral assessment

All animal behavioral testing was performed blinded to genotype. Mice were habituated to the testing room for at least 30 minutes before testing. Testing was performed in the same order for each animal over approximately 6 weeks (S1 Fig), beginning with the elevated zero maze assay, open-field test, social choice and preference for social novelty tests, high speed rotarod assay, circadian wheels assay, and resident-intruder test. Blood and brain tissues were collected afterwards for verification of genotypes.

### Elevated zero maze

The elevated zero maze was conducted as previously described [33,71,72]. It consists of a circular shaped, partially walled maze platform. Two opposite quadrants of the maze are enclosed whereas the other two are open. Mice were placed in one of the closed quadrants and their movement traced over the course of 5 min. The movement of the animals were recorded by a high-definition digital camera for offline analysis with video tracking software (ANY-maze, Stoelting Co.). Analysis included the quantification of percent of time spent in open arms and the number of entries. An entry was defined as 90% of the mouse body within a quadrant of the maze.

### Open-field test

Locomotor activity was measured similarly as previously described [33,71,72] via an open-field test where mice were individually placed into, and allowed to explore, a simple novel arena for a total of 15 min. Horizonal activity, vertical activity, and center activity was collected via infrared beam breaks. The 15 min trial was binned into 1 min epochs to assess habituation of activity as the animal became familiar to the arena.

### Social choice and preference for social novelty

The social choice test was carried out in a three-chambered apparatus, as previously described [33,71,72], that consisted of a center chamber and two end chambers. Before the start of the test and in a counter-balanced sequence, one end chamber was designated the social chamber, into which a stimulus mouse would be introduced, and the other end chamber was designed the nonsocial chamber. Two identical, clear Plexiglas cylinders with multiple holes to allow for air exchange were placed in each end chamber. In the habituation phase of the test (Phase 1), the experimental mouse freely explores the arena with empty cue cylinders in place for 10 min. In the social choice phase of the test (Phase 2), an age-matched stimulus mouse (M1) (adult, gonadectomized A/J mice) was placed in the cylinder in the social chamber while an inanimate object was simultaneously placed into the other cylinder in the nonsocial chamber. The social novelty phase immediately followed. In the social novelty phase (phase 3), the object used in phase 2 was replaced by a novel mouse (M2). The experimental mouse was tracked during a 10 min trial as it explores M2 and the familiarized mouse (M1) used in the choice phase. Image analysis software (ANY-maze) was used to determine the time of cue exploration and visits to each cue (snout of experimental mouse within 1 cm of cue cylinder) in all phases. The data was verified with manual video review and scoring.

### High speed rotarod

Mice were placed on an accelerating rotarod apparatus for 12 trials (three trials a day over four consecutive days) with at least 20 min of rest between the trials. On the first 2 consecutive days, mice were trained on the rod as it accelerated from 4–40 rpm, then on the next 2 days the rate was increased to 8–80 rpm [46]. Each trial lasted for a maximum of 5 min. Latency to fail in adjusting cadence (either falling from or grasping onto the rod for a full rotation) is recorded for each trial.

### Circadian wheels assay

Mice are single housed with free access to running wheels as previously described [36,73]. After habituation to the new housing conditions, wheel revolutions are collected to obtain a measure of voluntary wheel running in a 12 hr/12 hr light/dark cycle. After 14–16 days, mice are maintained in constant darkness to determine their endogenous period and phase shift, in addition to wheel running activity. Wheel revolutions and non-parametric analysis was obtained and analyzed with ClockLab software (Actimetrics, Willmette, IL) [36,74].

### Resident-intruder test

Similarly as previously described [75], mice were single housed for at least ten days to establish territoriality in their cages. Young intruder mice of the same sex, 6–8 weeks of age, were introduced into the experimental mouse cage followed by video recording for 10 min. Specific interactions by the resident experimental mouse are counted and timed in a 10 min trial. The specific interactions were classified as 1) Chasing/following with anogenital sniffing (cumulative time) and 2) Latency to the first aggressive behavior (grooming/barbering intruder mouse) and total duration of aggression.

### Tissue collection and quantitative RT-PCR analysis

Cortical tissues were rapidly dissected on ice and total RNAs were isolated using Trizol (Thermo Fisher) followed by a cleanup using the RNeasy plus micro kit (Qiagen cat. #74034). One microgram of RNA was converted into cDNA using oligo dT in the superscript III first-strand synthesis system (Thermo Fisher cat. # 18080051). Real-time-PCR was performed using power SYBR green PCR master mix (Applied Biosystems, cat. #4367659). The primers used in the quantitative PCR were as following: Nrxn1α, exon 9F 5’-GAGATGCTGGATGGCCACTT-3’ and exon 10R 5’-GGGAGTGCGTAGTGTGTTGA-3’; Nrxn1β, exon1F 5-CATGGCAGCAGCAAGCATCA-3’ and exon 2R 5-AATCTGTCCACCACCTTTGC-3’; Nrxn1γ, exon 1F 5’-GATGGCACTGTGAAAACTCGC-3’ and exon 2R 5’-CTCACAGGGGTCAATGTCCT-3’; Gapdh, forward primer 5’-TGTCAAGCTCATTTCCTGGTGTGA-3’ and reverse primer 5’-TCTTACTCCTTGGAGGCCATGT-3’. Results were quantified on an ABI 7900 system. All RNA expression levels were normalized to Gapdh using the ΔΔCT method.

### Statistics

For behavioral assays, we chose similar sample sizes for all behavioral experiments. The number of mice used in each group was predetermined based on our previous studies [33,71,72]. All data sets were analyzed using the Shapiro-Wilk test for normality. For 2-sample comparisons, data sets with normal distributions were analyzed for significance using the unpaired t test, whereas data sets with non-normal distributions were analyzed using the Mann-Whitney U test. For multiple-comparisons, ordinary one-way ANOVA was performed with Tukey’s multiple-comparison tests. For high speed rotarod test, two-way repeated measures ANOVA was conducted for the appropriate data sets with Dunnett’s multiple-comparison tests. The quantitative RT-PCR results were analyzed using one-way ANOVA. All tests were 2-tailed.

## Supporting information

S1 Fig The Schema of the behavioral testing. The diagram outlines the schedule of behavioral testing for *Nrxn1* mutant and control mice.
(TIF)

S2 Fig ΔExon9 and ΔExon1 mice show normal locomotor activity and anxiety-related behaviors. **A-D.** ΔExon9/+, ΔExon9/ΔExon9 and ΔExon1/+mice show normal anxiety-related behaviors in the EZM test in the time spending in the open arms and the number of entries to the open arms in males (A,C) and females (B,D). Male +/+, n = 14; male ΔExon9/+, n = 18; male ΔExon9/ΔExon9, n = 9; female ΔExon9/+, n = 18; female ΔExon9/+, n = 17; female ΔExon9/ΔExon9, n = 11 in the exon 9 deletion studies. The male +/+, n = 17; male ΔExon1/+, n = 11; female +/+, n = 12; female ΔExon1/+, n = 11 in the exon 1 deletion studies. **E-L.** ΔExon9/+ and ΔExon9/ΔExon9 mice show normal locomotor activity in the open field test (X,Y-axis beam breaks, %Center beam breaks, and X,Y-axis beam breaks over time) in both males (E-F) and females (I-J). ΔExon1/+mice male mice exhibit slightly increased overall X,Y-axis beam breaks compared to +/+ (G), but no significant differences in %Center beam breaks or beam breaks over time (H). Female ΔExon1/+ mice show normal locomotor activity compared to +/+ (X,Y-axis beam breaks, %Center beam breaks, and X,Y-axis beam breaks over time; K-L). One-way ANOVA test with Tukey’s multiple comparison test was used to analyze the ΔExon9 data and Mann-Whitney U test was used to analyze the ΔExon1 data, **p<0.01. The number of animals used in these tests is the same as those in (A-D). Data are represented as mean ± SEM in all graphs.
(TIF)

S3 Fig Mice carrying ΔIntron17 show normal locomotor activity and anxiety-related behaviors. **A-B.** ΔIntron17/+ and ΔIntron17/ΔIntron17 mice show similar behaviors compared to wild type (+/+) controls in the elevated zero maze (EZM) test, males (A) and females (B), for time spent in the open arms and total number of open arm entries. Male *+/+*, n = 10; male ΔIntron17/+, n = 16; male ΔIntron17/ΔIntron17, n = 13; female *+/+*, n = 13; female ΔIntron17/+, n = 14; female ΔIntron17/ΔIntron17, n = 12. **C-D.** ΔIntron17/+ and ΔIntron17/ΔIntron17 mice show similar behavior compared to the +/+ mice in the open field test for X,Y-axis beam breaks and %Center beam breaks in both males (C) and females (D). The number of animals used in the test is the same as in (A-B). **E-F.** X,Y-axis beam breaks over time in +/+, ΔIntron17/+, and ΔIntron17/ΔIntron17 male (E) and female (F) mice show similar locomotor activity behavior across all groups in the open field test. The number of animals used in these tests is the same as those in (A-B). One-way ANOVA test with Tukey’s multiple comparison test was used to analyze the data. Data are represented as mean ± SEM in all graphs.
(TIF)

S4 Fig Mice carrying ΔIntron17 show normal sociability in the social approach test. **A-D.** No genotype difference was observed in time spent sniffing the social cylinder among ΔIntron17 males (A) and females (C), as well as sniffing the novel mouse among ΔIntron17 males (B) and females (D). The number of animals used in each group in these tests are the same as those in S3 Fig. One-way ANOVA test with Tukey’s multiple comparison test was used to analyze the data. Data are represented as mean ± SEM in all graphs.
(TIF)

S5 Fig Mice carrying ΔIntron17 show normal social behaviors in the Resident-intruder test. **A-D.** No genotype difference was observed in time spent sniffing and chasing the intruder mouse (A), exhibiting aggressive behaviors (B), or attack latency (C) among ΔIntron17 males. ΔIntron17 female mice show no genotype difference in time spent sniffing and chasing the intruder mouse (D). The number of animals used in each group in these tests are the same as those in S3 Fig. One-way ANOVA test with Tukey’s multiple comparison test was used to analyze the data. Data are represented as mean ± SEM in all graphs.
(TIF)

S6 Fig Non-parametric measurements of rest-activity traits in L/D and D/D phase in ΔExon9 and ΔExon1 mouse models. **A-B.** Average activity in the most active 10 hours of the day in the L/D (A) and D/D (B) phase in +/+, ΔExon9/+, ΔExon9/ΔExon9 animals, males (left) and females (right). **C.** Intradaily Variability in the D/D phase in +/+, ΔExon9/+, ΔExon9/ΔExon9 animals. Kruskal-Wallis test with Dunn’s multiple comparison test was used to analyze the ΔExon9 results (A-C). **D-E.** Average activity in the most active 10 hours of the day in the L/D (D) and D/D (E) phase in +/+ and ΔExon1/+ animals. Unpaired t test was used in (D) and Mann-Whitney U test was used in (E). **F.** Intradaily Variability in the D/D phase in +/+ and ΔExon1/+ animals. Mann-Whitney U test was used to analyze (F). The number of animals used in the test is the same as in S2 Fig. *p<0.05; **p<0.01; ***p<0.001; ****p<0.0001. Data are represented as mean ± SEM in all graphs.
(TIF)

S7 Fig Additional non-parametric measurements of rest-activity traits in L/D and D/D phase in ΔExon9 and ΔExon1 mouse models. **A-B.** Average activity in the least active 5 hours of the day in the L/D (A) and D/D (B) phase in +/+, ΔExon9/+, ΔExon9/ΔExon9 animals, males (left) and females (right). **C-D.** Start time of the least active 5 hours of the day the L/D (C) and D/D (D) phase in +/+, ΔExon9/+, ΔExon9/ΔExon9 animals. **E-F.** Start time of the most active 10 hours of the day the LD (E) and DD (F) phase in +/+, ΔExon9/+, ΔExon9/ΔExon9 animals. **G-H.** Average activity in the least active 5 hours of the day in the L/D (G) and D/D (H) phase in +/+ and ΔExon1/+ animals; male and female ΔExon1/+ mice exhibited reduced activity during L5 compared to +/+. **I-J.** Start time of the least active 5 hours of the day the L/D (I) and D/D (J) phase in +/+ and ΔExon1/+ animals. **K-L.** Start time of the most active 10 hours of the day the L/D (K) and D/D (L) phase in +/+ and ΔExon1/+ animals. The number of animals used in the test is the same as in S2 Fig. Kruskal-Wallis test with Dunn’s multiple comparison test was used to analyze the ΔExon9 data (A-F). Mann-Whitney U test was used to analyze the ΔExon1 data (G-L). *p<0.05; ***p<0.001. Data are represented as mean ± SEM in all graphs.
(TIF)

S8 Fig Activity bout assessment in the D/D phase in ΔExon9 and ΔExon1 mouse models. **A.** Bouts per day in the D/D phase in +/+, ΔExon9/+, ΔExon9/ΔExon9 animals. **B.** Bout length in the D/D phase in +/+, ΔExon9/+, ΔExon9/ΔExon9 animals. **C.** Revolutions per bout in the D/D phase in +/+, ΔExon9/+, ΔExon9/ΔExon9 animals. Kruskal-Wallis test with Dunn’s multiple comparison test was used to analyze the ΔExon9 data in (A-C). **D.** Bouts per day in the D/D phase in +/+ and ΔExon1/+ animals. **E.** Bout length in the D/D phase in +/+ and ΔExon1/+ animals. **F.** Revolutions per bout in the D/D phase in +/+ and ΔExon1/+ animals. The number of animals used in the test is the same as in S2 Fig. In (D-F), if groups were normally distributed (D’Agostino & Pearson test) and didn’t have significantly different variance (F test), the unpaired t test was used. If samples were not equally distributed or had different variances, Mann-Whitney U test was used. *p<0.05; **p<0.01; ***p<0.001; ****p<0.0001. Data are represented as mean ± SEM in all graphs.
(TIF)

S9 Fig Activity assessment in ΔIntron17 mice. **A-B.** Representative actograms from male (A) and female (B) mice under two different lighting conditions: (1) 12 h of light (indicated by the yellow shading), 12 h of dark (L/D) and (2) constant dark (D/D). Actograms depict locomotor activity (wheel revolutions) across time with days stacked vertically and double plotted with the x axis spanning 2 days (48 hours). Grey squares indicate activity onsets. **C.** Activity profiles averaged over 5 consecutive days of L/D conditions across sex and genotype. **D.** Endogenous period across sex and genotype. Mean endogenous periods per group: male +/+, 23.61; male ΔIntron17/+, 23.67; male ΔIntron17/ΔIntron17, 23.44; female +/+, 23.63; female ΔIntron17/+, 23.68; and female ΔIntron17/ΔIntron17, 23.65. **E.** Phase shift across sex and genotype. **F.** Intradaily variability in the L/D phase across sex and genotype. **G.** Intradaily variability in the D/D phase across sex and genotype. **H.** Interdaily stability in the L/D phase across sex and genotype. **I-J.** Average activity in the most active 10 hours of the day in the L/D (I) and D/D (J) phase. **K-L.** Start time of the most active 10 hours of the day the L/D (K) and D/D (L) phase. **M-N.** Average activity in the least active 5 hours of the day in the L/D (M) and D/D (N) phase. **O-P.** Start time of the least active 5 hours of the day the L/D (O) and D/D (P) phase. The number of animals used in the test is the same as in S3 Fig. If groups were normally distributed (D’Agostino & Pearson test) and didn’t have significantly different variance (F test), Ordinary one-way ANOVA test with Tukey’s multiple comparison test was used. If samples were not equally distributed or had different variances, Kruskal-Wallis test with Dunn’s multiple comparison test was used. Data are represented as mean ± SEM in all graphs.
(TIF)

S10 Fig Activity bout assessment in the L/D and D/D phase in ΔIntron17 mice. **A-B.** Representative actograms with activity indicated in green and bouts marked with blue squares from male (A) and female (B) mice under two different lighting condition: (1) 12 h of light (indicated by the yellow shading), 12 h of dark (L/D) and (2) constant dark (D/D). Actograms depict locomotor activity (wheel revolutions) in green across time with days stacked vertically and double plotted with the x axis spanning 2 days (48 hours). **C.** Bouts per day in the L/D (top) and D/D (bottom) phase. **D.** Bout length in the L/D (top) and D/D (bottom) phase. **E.** Revolutions per bout in the L/D phase. **F.** Revolutions per bout in the D/D phase. The number of animals used in the test is the same as in S3 Fig. If groups were normally distributed (D’Agostino & Pearson test) and didn’t have significantly different variance (F test), Ordinary one-way ANOVA test with Tukey’s multiple comparison test was used. If samples were not equally distributed or had different variances, Kruskal-Wallis test with Dunn’s multiple comparison test was used. Data are represented as mean ± SEM in all graphs.
(TIF)

S11 Fig Mice carrying ΔIntron17 show normal motor skill learning and motor coordination. **A**. Time the *+/+*, ΔIntron17/+ and ΔIntron17/ΔIntron17 male mice stay on accelerating rotarod (4–40 rpm in 5 min). Mice were tested 3 trials per day for 2 consecutive days. **B.** Time the same mice stay on a higher accelerating rotarod (8–80 rpm in 5 min). Mice were tested 3 trials per day for the next 2 consecutive days. **C.** Time the *+/+*, ΔIntron17/+ and ΔIntron17/ΔIntron17 female mice stay on accelerating rotarod (4–40 rpm in 5 min). Mice were tested 3 trials per day for 2 consecutive days. **D.** Time the same female mice stay on a higher accelerating rotarod (8–80 rpm in 5 min). Mice were tested 3 trials per day for the next 2 consecutive days. The number of animals used in each group in the high speed rotarod is the same as in the S3 Fig. Two-way repeat measure ANOVA test with Dunnett’s multiple comparison test was used to analyze the data. Data are represented as mean ± SEM in all graphs.
(TIF)

S1 Table Statistical analyses of behavioral phenotypes in the context of sex and genotypes for *Nrxn1* Exon9 deletion mouse model (+/+, ΔExon9/+, ΔExon9/ΔExon9). (PDF)

S2 Table Statistical analyses of behavioral phenotypes in the context of sex and genotypes for *Nrxn1* Exon1 deletion mouse model (+/+, ΔExon1/+). (PDF)

S3 Table Statistical analyses of behavioral phenotypes in the context of sex and genotypes for *Nrxn1* Intron17 deletion mouse model (+/+, ΔIntron17/+, ΔIntron17/ΔIntron17). (PDF)
