# Supplementary figures and images for: Allelic contribution of Nrxn1α to autism-relevant behavioral phenotypes in mice
Source: PLoS Genet. 2023 Feb 27;19(2):e1010659. doi: 10.1371/journal.pgen.1010659 (PMC9997995; doi:10.1371/journal.pgen.1010659)

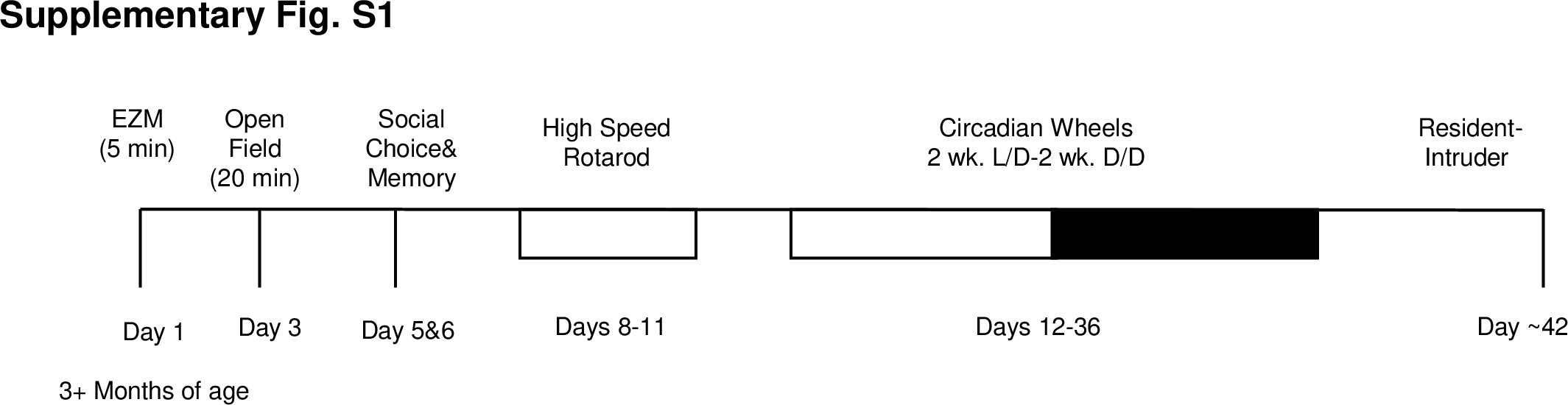

Supplement: S1 Fig — The diagram outlines the schedule of behavioral testing for Nrxn1 mutant and control mice. (TIF) [file pgen.1010659.s001.tif]

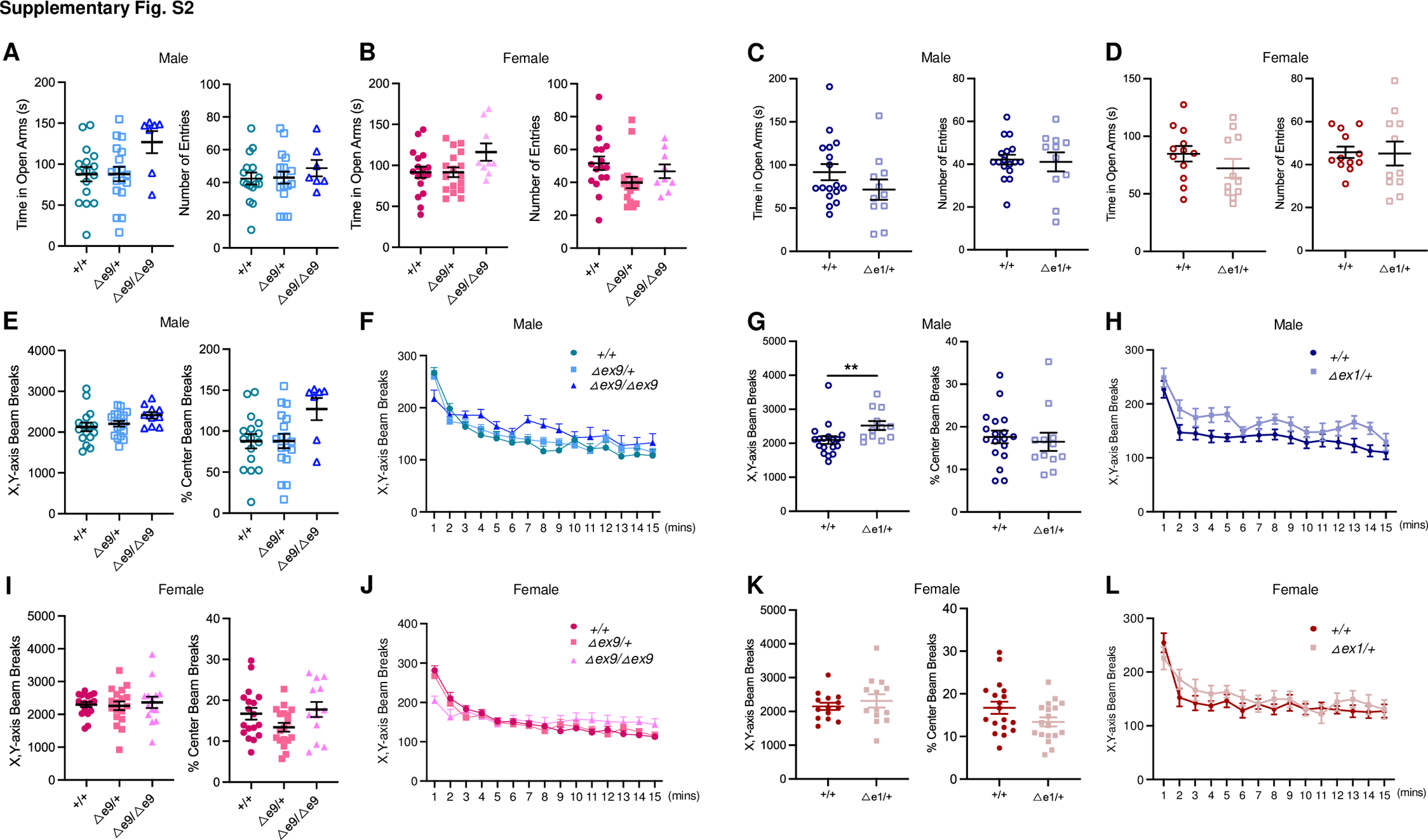

Supplement: S2 Fig — A-D. ΔExon9/+, ΔExon9/ΔExon9 and ΔExon1/+mice show normal anxiety-related behaviors in the EZM test in the time spending in the open arms and the number of entries to the open arms in males (A,C) and females (B,D). Male +/+, n = 14; male ΔExon9/+, n = 18; male ΔExon9/ΔExon9, n = 9; female ΔExon9/+, n = 18; female ΔExon9/+, n = 17; female ΔExon9/ΔExon9, n = 11 in the exon 9 deletion studies. The male +/+, n = 17; male ΔExon1/+, n = 11; female +/+, n = 12; female ΔExon1/+, n = 11 in the exon 1 deletion studies. E-L. ΔExon9/+ and ΔExon9/ΔExon9 mice show normal locomotor activity in the open field test (X,Y-axis beam breaks, %Center beam breaks, and X,Y-axis beam breaks over time) in both males (E-F) and females (I-J). ΔExon1/+mice male mice exhibit slightly increased overall X,Y-axis beam breaks compared to +/+ (G), but no significant differences in %Center beam breaks or beam breaks over time (H). Female ΔExon1/+ mice show normal locomotor activity compared to +/+ (X,Y-axis beam breaks, %Center beam breaks, and X,Y-axis beam breaks over time; K-L). One-way ANOVA test with Tukey’s multiple comparison test was used to analyze the ΔExon9 data and Mann-Whitney U test was used to analyze the ΔExon1 data, **p<0.01. The number of animals used in these tests is the same as those in (A-D). Data are represented as mean ± SEM in all graphs. (TIF) [file pgen.1010659.s002.tif]

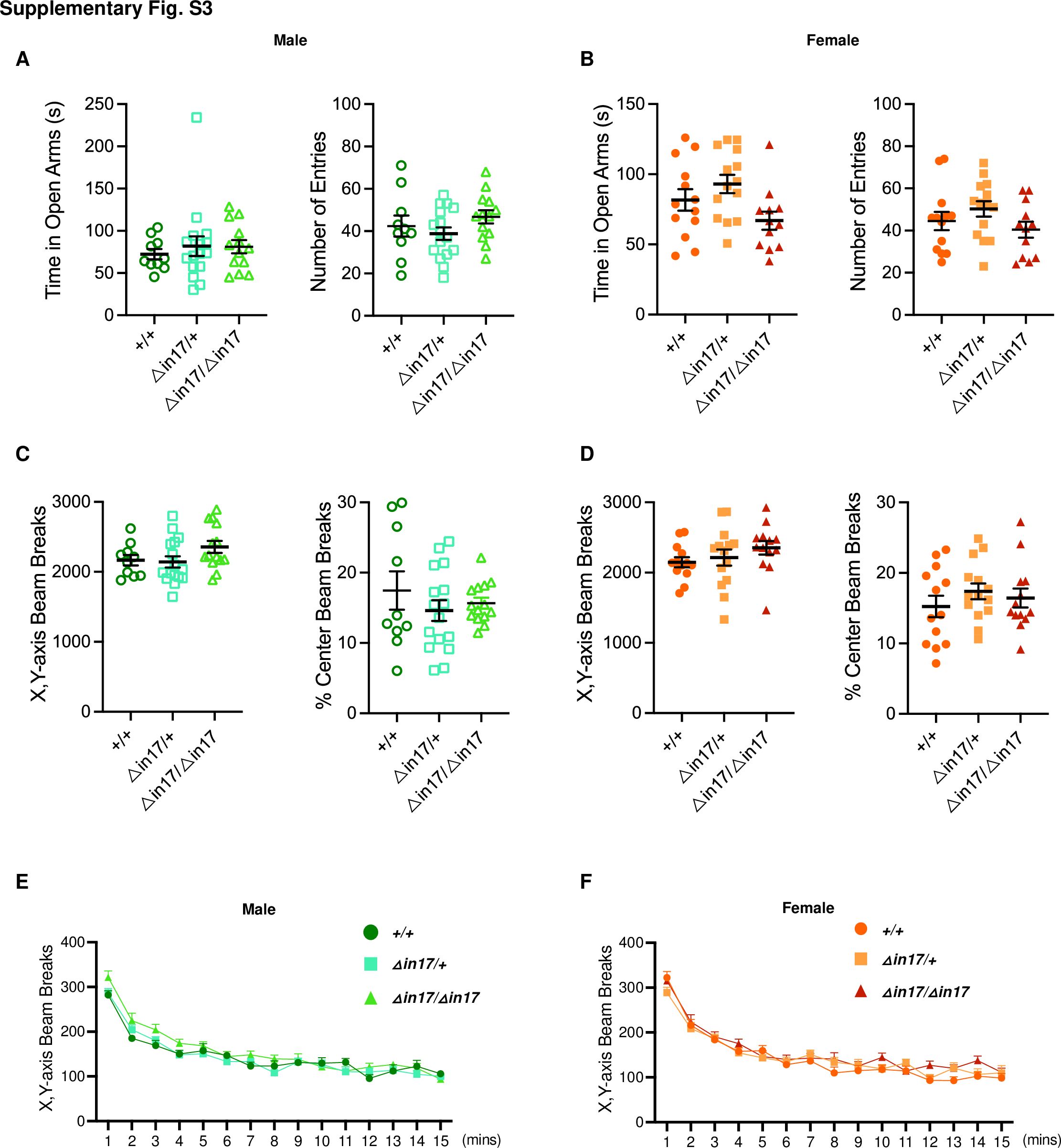

Supplement: S3 Fig — A-B. ΔIntron17/+ and ΔIntron17/ΔIntron17 mice show similar behaviors compared to wild type (+/+) controls in the elevated zero maze (EZM) test, males (A) and females (B), for time spent in the open arms and total number of open arm entries. Male +/+, n = 10; male ΔIntron17/+, n = 16; male ΔIntron17/ΔIntron17, n = 13; female +/+, n = 13; female ΔIntron17/+, n = 14; female ΔIntron17/ΔIntron17, n = 12. C-D. ΔIntron17/+ and ΔIntron17/ΔIntron17 mice show similar behavior compared to the +/+ mice in the open field test for X,Y-axis beam breaks and %Center beam breaks in both males (C) and females (D). The number of animals used in the test is the same as in (A-B). E-F. X,Y-axis beam breaks over time in +/+, ΔIntron17/+, and ΔIntron17/ΔIntron17 male (E) and female (F) mice show similar locomotor activity behavior across all groups in the open field test. The number of animals used in these tests is the same as those in (A-B). One-way ANOVA test with Tukey’s multiple comparison test was used to analyze the data. Data are represented as mean ± SEM in all graphs. (TIF) [file pgen.1010659.s003.tif]

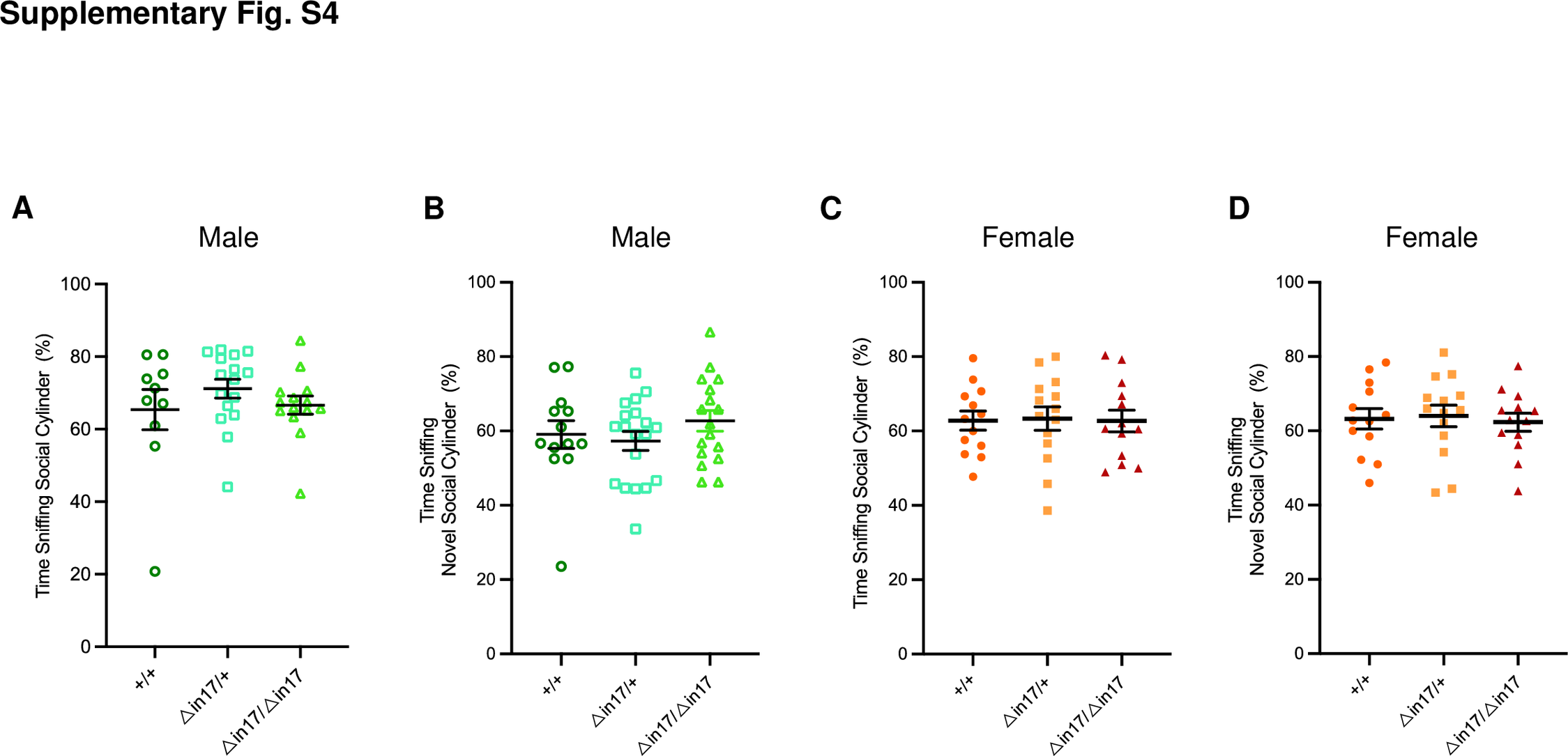

Supplement: S4 Fig — A-D. No genotype difference was observed in time spent sniffing the social cylinder among ΔIntron17 males (A) and females (C), as well as sniffing the novel mouse among ΔIntron17 males (B) and females (D). The number of animals used in each group in these tests are the same as those in S3 Fig. One-way ANOVA test with Tukey’s multiple comparison test was used to analyze the data. Data are represented as mean ± SEM in all graphs. (TIF) [file pgen.1010659.s004.tif]

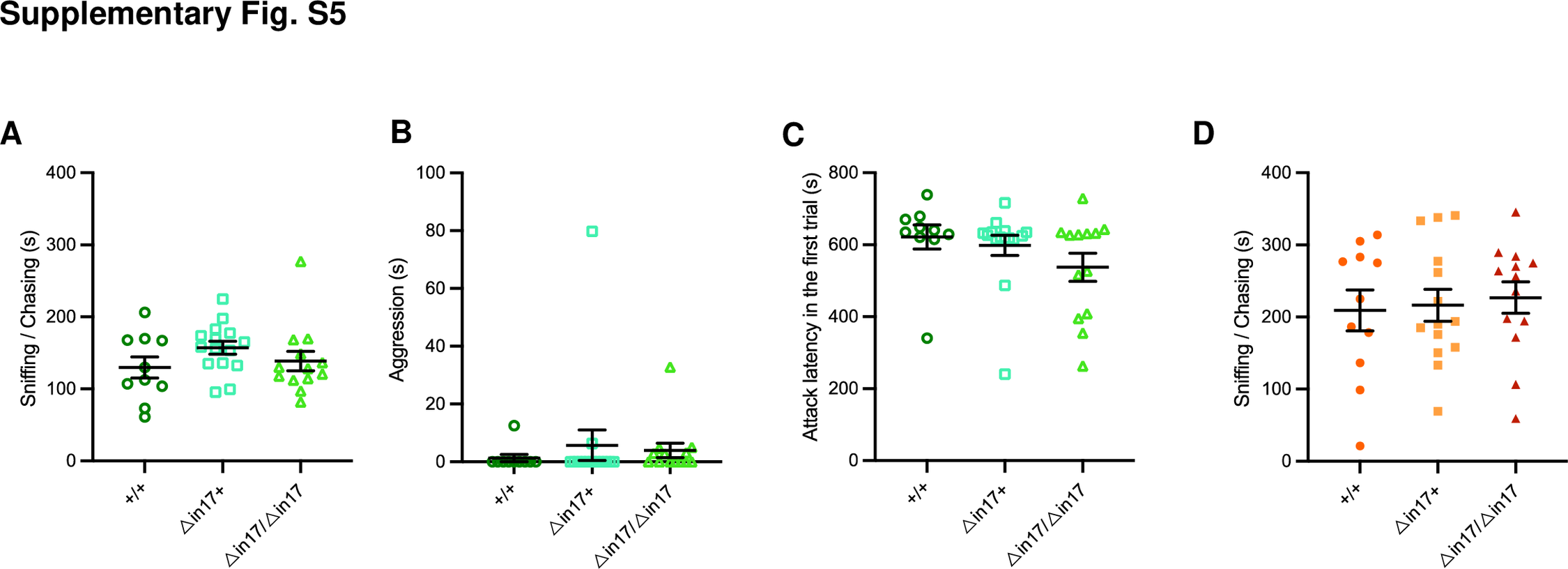

Supplement: S5 Fig — A-D. No genotype difference was observed in time spent sniffing and chasing the intruder mouse (A), exhibiting aggressive behaviors (B), or attack latency (C) among ΔIntron17 males. ΔIntron17 female mice show no genotype difference in time spent sniffing and chasing the intruder mouse (D). The number of animals used in each group in these tests are the same as those in S3 Fig. One-way ANOVA test with Tukey’s multiple comparison test was used to analyze the data. Data are represented as mean ± SEM in all graphs. (TIF) [file pgen.1010659.s005.tif]

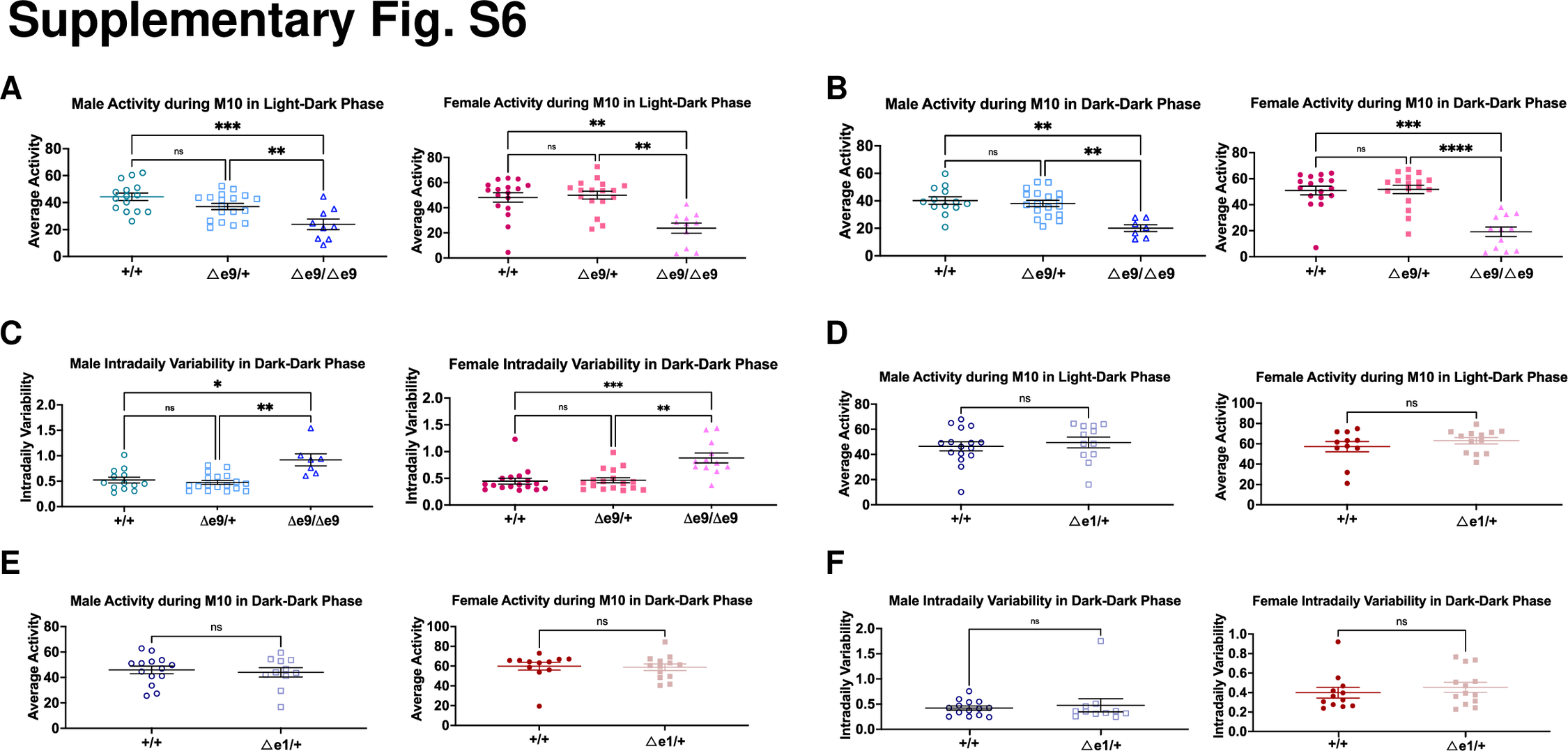

Supplement: S6 Fig — A-B. Average activity in the most active 10 hours of the day in the L/D (A) and D/D (B) phase in +/+, ΔExon9/+, ΔExon9/ΔExon9 animals, males (left) and females (right). C. Intradaily Variability in the D/D phase in +/+, ΔExon9/+, ΔExon9/ΔExon9 animals. Kruskal-Wallis test with Dunn’s multiple comparison test was used to analyze the ΔExon9 results (A-C). D-E. Average activity in the most active 10 hours of the day in the L/D (D) and D/D (E) phase in +/+ and ΔExon1/+ animals. Unpaired t test was used in (D) and Mann-Whitney U test was used in (E). F. Intradaily Variability in the D/D phase in +/+ and ΔExon1/+ animals. Mann-Whitney U test was used to analyze (F). The number of animals used in the test is the same as in S2 Fig. *p<0.05; **p<0.01; ***p<0.001; ****p<0.0001. Data are represented as mean ± SEM in all graphs. (TIF) [file pgen.1010659.s006.tif]

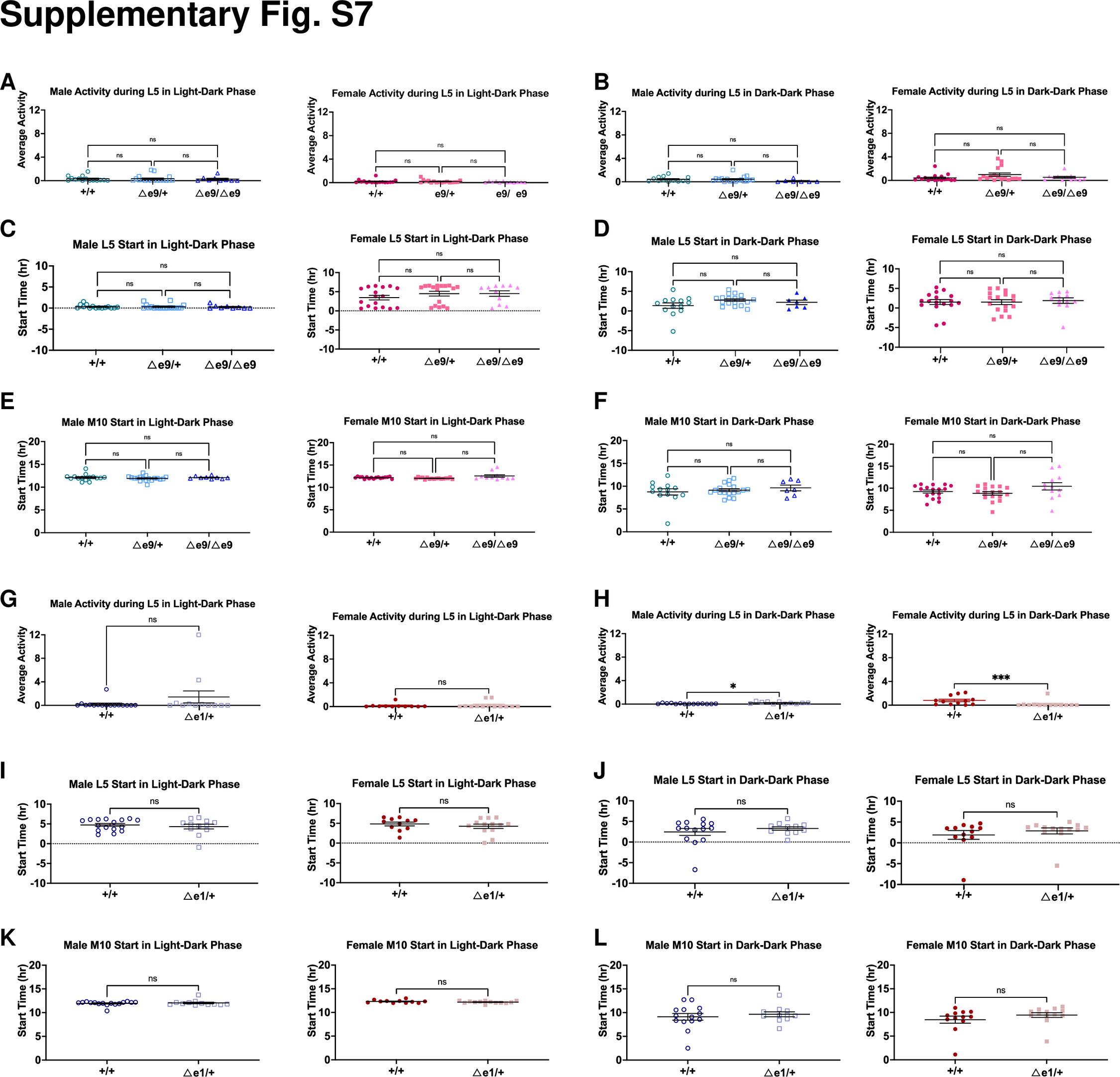

Supplement: S7 Fig — A-B. Average activity in the least active 5 hours of the day in the L/D (A) and D/D (B) phase in +/+, ΔExon9/+, ΔExon9/ΔExon9 animals, males (left) and females (right). C-D. Start time of the least active 5 hours of the day the L/D (C) and D/D (D) phase in +/+, ΔExon9/+, ΔExon9/ΔExon9 animals. E-F. Start time of the most active 10 hours of the day the LD (E) and DD (F) phase in +/+, ΔExon9/+, ΔExon9/ΔExon9 animals. G-H. Average activity in the least active 5 hours of the day in the L/D (G) and D/D (H) phase in +/+ and ΔExon1/+ animals; male and female ΔExon1/+ mice exhibited reduced activity during L5 compared to +/+. I-J. Start time of the least active 5 hours of the day the L/D (I) and D/D (J) phase in +/+ and ΔExon1/+ animals. K-L. Start time of the most active 10 hours of the day the L/D (K) and D/D (L) phase in +/+ and ΔExon1/+ animals. The number of animals used in the test is the same as in S2 Fig. Kruskal-Wallis test with Dunn’s multiple comparison test was used to analyze the ΔExon9 data (A-F). Mann-Whitney U test was used to analyze the ΔExon1 data (G-L). *p<0.05; ***p<0.001. Data are represented as mean ± SEM in all graphs. (TIF) [file pgen.1010659.s007.tif]

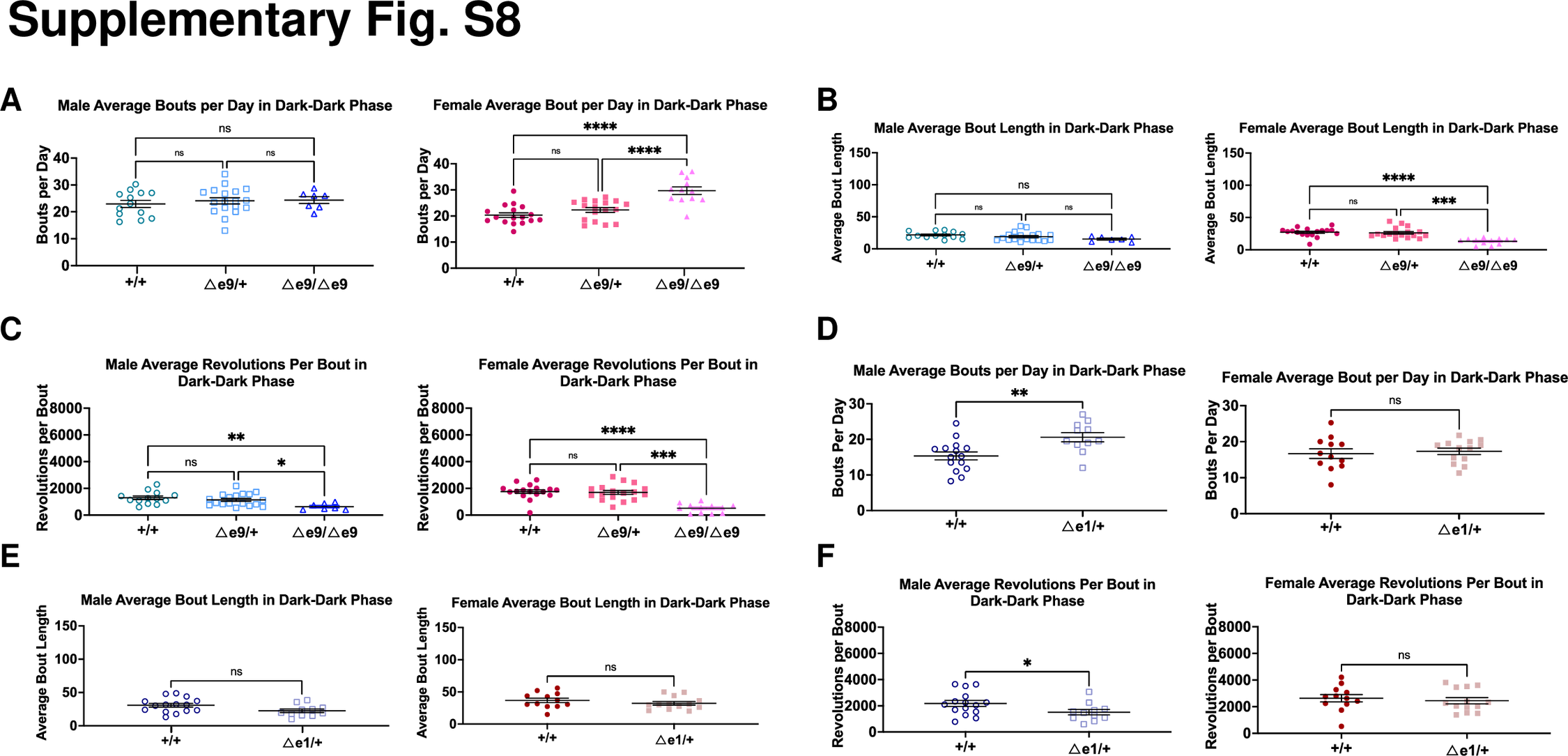

Supplement: S8 Fig — A. Bouts per day in the D/D phase in +/+, ΔExon9/+, ΔExon9/ΔExon9 animals. B. Bout length in the D/D phase in +/+, ΔExon9/+, ΔExon9/ΔExon9 animals. C. Revolutions per bout in the D/D phase in +/+, ΔExon9/+, ΔExon9/ΔExon9 animals. Kruskal-Wallis test with Dunn’s multiple comparison test was used to analyze the ΔExon9 data in (A-C). D. Bouts per day in the D/D phase in +/+ and ΔExon1/+ animals. E. Bout length in the D/D phase in +/+ and ΔExon1/+ animals. F. Revolutions per bout in the D/D phase in +/+ and ΔExon1/+ animals. The number of animals used in the test is the same as in S2 Fig. In (D-F), if groups were normally distributed (D’Agostino & Pearson test) and didn’t have significantly different variance (F test), the unpaired t test was used. If samples were not equally distributed or had different variances, Mann-Whitney U test was used. *p<0.05; **p<0.01; ***p<0.001; ****p<0.0001. Data are represented as mean ± SEM in all graphs. (TIF) [file pgen.1010659.s008.tif]

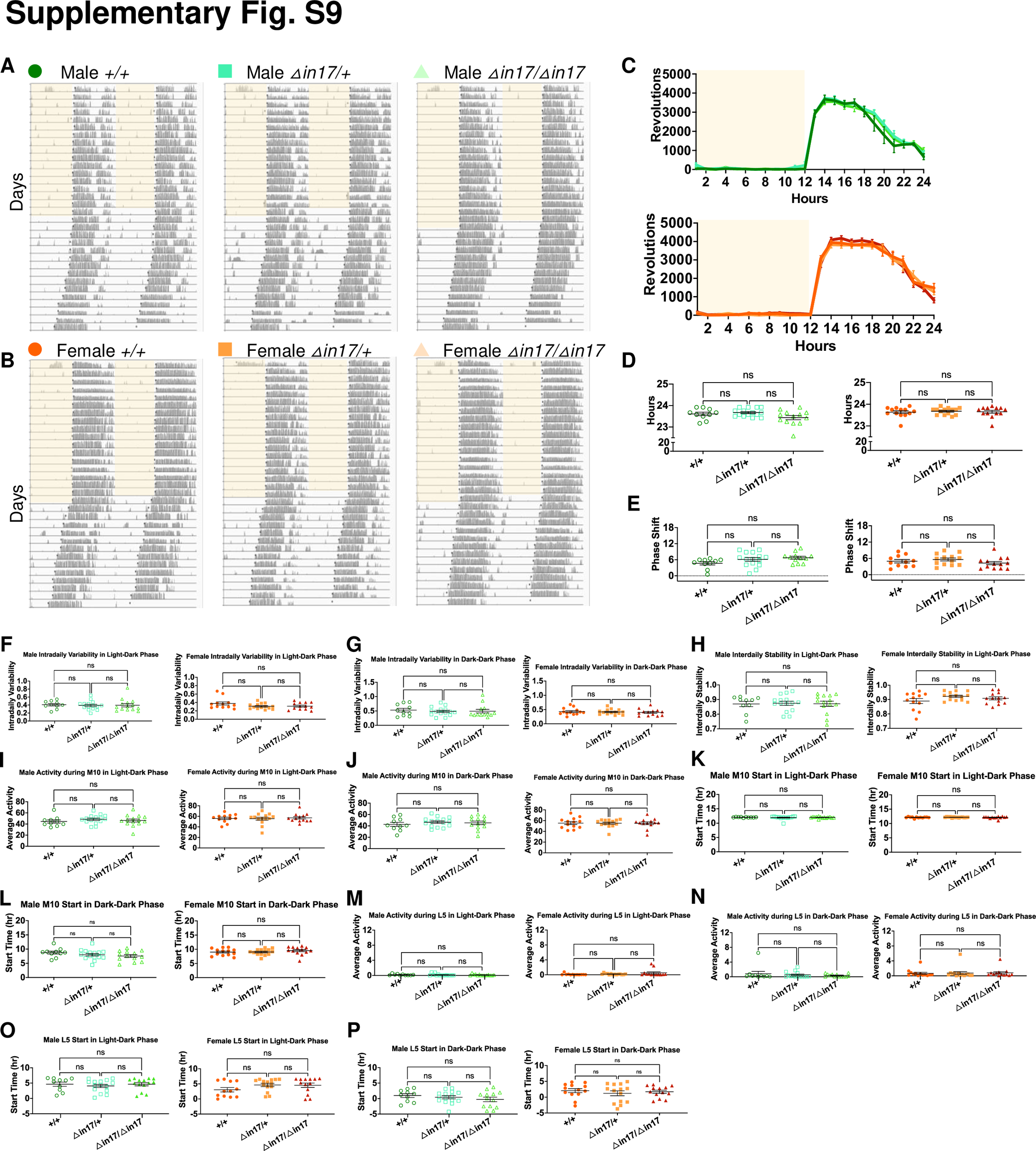

Supplement: S9 Fig — A-B. Representative actograms from male (A) and female (B) mice under two different lighting conditions: (1) 12 h of light (indicated by the yellow shading), 12 h of dark (L/D) and (2) constant dark (D/D). Actograms depict locomotor activity (wheel revolutions) across time with days stacked vertically and double plotted with the x axis spanning 2 days (48 hours). Grey squares indicate activity onsets. C. Activity profiles averaged over 5 consecutive days of L/D conditions across sex and genotype. D. Endogenous period across sex and genotype. Mean endogenous periods per group: male +/+, 23.61; male ΔIntron17/+, 23.67; male ΔIntron17/ΔIntron17, 23.44; female +/+, 23.63; female ΔIntron17/+, 23.68; and female ΔIntron17/ΔIntron17, 23.65. E. Phase shift across sex and genotype. F. Intradaily variability in the L/D phase across sex and genotype. G. Intradaily variability in the D/D phase across sex and genotype. H. Interdaily stability in the L/D phase across sex and genotype. I-J. Average activity in the most active 10 hours of the day in the L/D (I) and D/D (J) phase. K-L. Start time of the most active 10 hours of the day the L/D (K) and D/D (L) phase. M-N. Average activity in the least active 5 hours of the day in the L/D (M) and D/D (N) phase. O-P. Start time of the least active 5 hours of the day the L/D (O) and D/D (P) phase. The number of animals used in the test is the same as in S3 Fig. If groups were normally distributed (D’Agostino & Pearson test) and didn’t have significantly different variance (F test), Ordinary one-way ANOVA test with Tukey’s multiple comparison test was used. If samples were not equally distributed or had different variances, Kruskal-Wallis test with Dunn’s multiple comparison test was used. Data are represented as mean ± SEM in all graphs. (TIF) [file pgen.1010659.s009.tif]

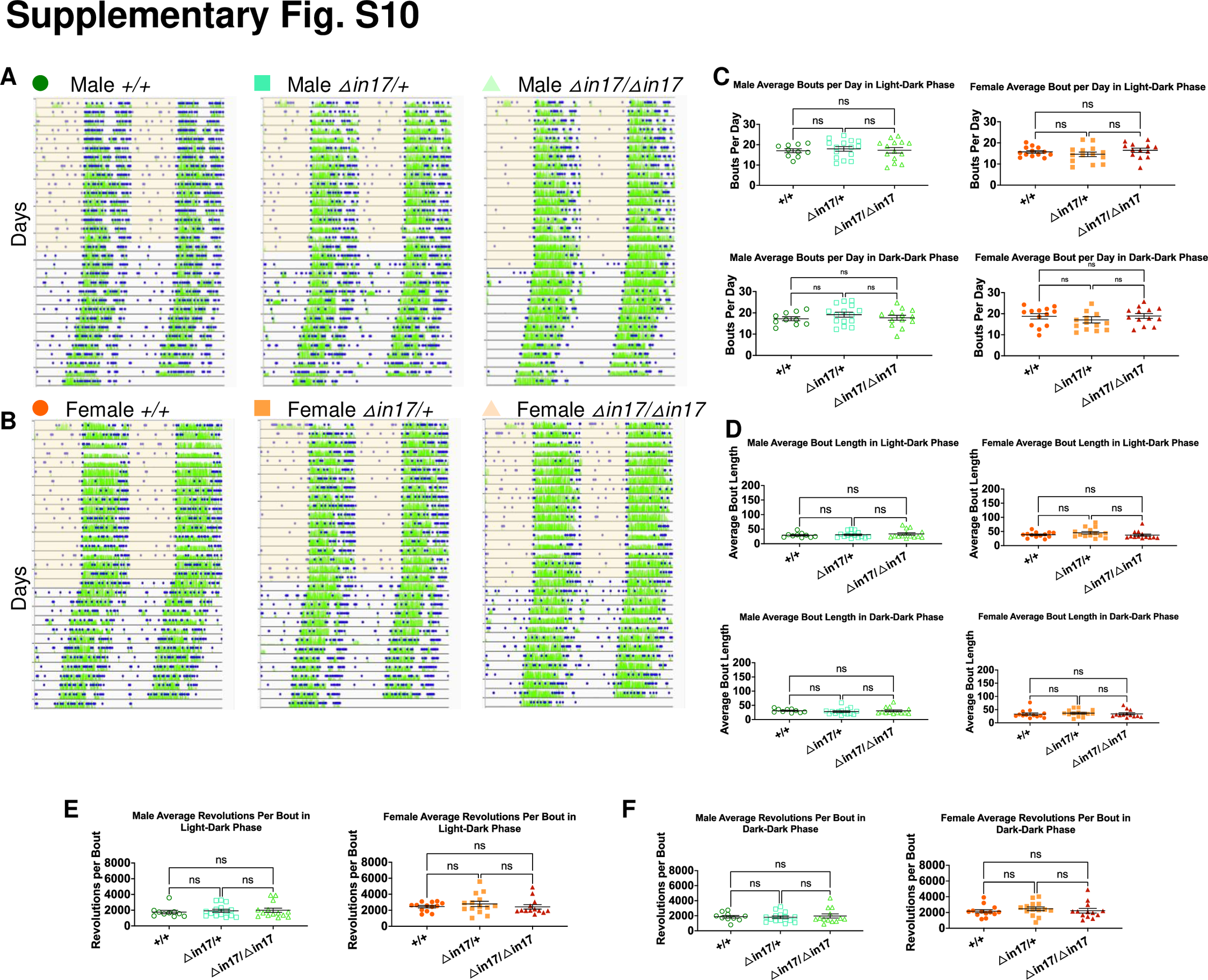

Supplement: S10 Fig — A-B. Representative actograms with activity indicated in green and bouts marked with blue squares from male (A) and female (B) mice under two different lighting condition: (1) 12 h of light (indicated by the yellow shading), 12 h of dark (L/D) and (2) constant dark (D/D). Actograms depict locomotor activity (wheel revolutions) in green across time with days stacked vertically and double plotted with the x axis spanning 2 days (48 hours). C. Bouts per day in the L/D (top) and D/D (bottom) phase. D. Bout length in the L/D (top) and D/D (bottom) phase. E. Revolutions per bout in the L/D phase. F. Revolutions per bout in the D/D phase. The number of animals used in the test is the same as in S3 Fig. If groups were normally distributed (D’Agostino & Pearson test) and didn’t have significantly different variance (F test), Ordinary one-way ANOVA test with Tukey’s multiple comparison test was used. If samples were not equally distributed or had different variances, Kruskal-Wallis test with Dunn’s multiple comparison test was used. Data are represented as mean ± SEM in all graphs. (TIF) [file pgen.1010659.s010.tif]

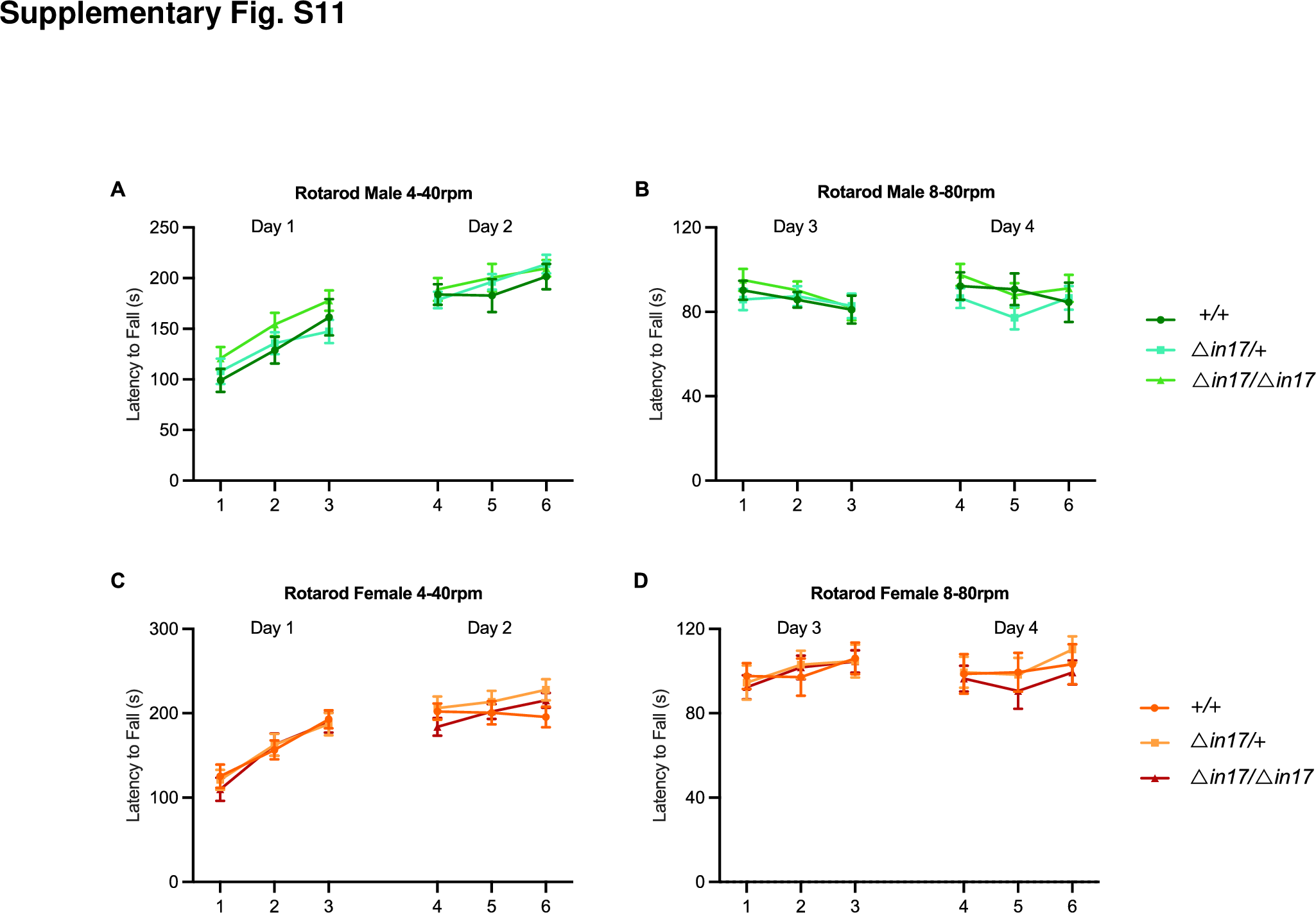

Supplement: S11 Fig — A. Time the +/+, ΔIntron17/+ and ΔIntron17/ΔIntron17 male mice stay on accelerating rotarod (4–40 rpm in 5 min). Mice were tested 3 trials per day for 2 consecutive days. B. Time the same mice stay on a higher accelerating rotarod (8–80 rpm in 5 min). Mice were tested 3 trials per day for the next 2 consecutive days. C. Time the +/+, ΔIntron17/+ and ΔIntron17/ΔIntron17 female mice stay on accelerating rotarod (4–40 rpm in 5 min). Mice were tested 3 trials per day for 2 consecutive days. D. Time the same female mice stay on a higher accelerating rotarod (8–80 rpm in 5 min). Mice were tested 3 trials per day for the next 2 consecutive days. The number of animals used in each group in the high speed rotarod is the same as in the S3 Fig. Two-way repeat measure ANOVA test with Dunnett’s multiple comparison test was used to analyze the data. Data are represented as mean ± SEM in all graphs. (TIF) [file pgen.1010659.s011.tif]
