# Supplementary material for: Allelic contribution of Nrxn1α to autism-relevant behavioral phenotypes in mice
Source: PLoS Genet. 2023 Feb 27;19(2):e1010659. doi: 10.1371/journal.pgen.1010659 (PMC9997995; doi:10.1371/journal.pgen.1010659)
Supplement: S1 Table — (PDF) [file pgen.1010659.s012.pdf]

**Supplementary Table S1 – Statistical analyses of behavioral phenotypes in the context of sex and genotypes for *Nrxn1* Exon9 deletion mouse model (+/+, ΔExon9/+, ΔExon9/ΔExon9)**

| Behavioral Test | Parameter | Comparison | Results |
|---|---|---|---|
| **3-Box Social interaction Test** | | | |
| Preference Test for Social interaction | Time with social cylinder | Sex and Genotype | Mixed-effect analysis; No main effect of Sex: F (1, 81) = 1.594, P=0.2104;No main effect of Genotype: F(2, 81)= 1.314, P=0.2743; No Sex x Genotype interaction: F(2, 81) = 0.3121, P=0.7328. |
| Preference Test for Novel Animal | Time with novel adult mouse | Sex and Genotype | Mixed-effect analysis; No main effect of Sex: F(1,34) =0.7845, P=0.3820; **Significant effect of Genotype: F(2,47)=4.458, P<0.05; Significant effect of Sex X Genotype interaction: F(2,47)=3.804, P<0.05.** |
| **Resident-intruder Test** | | | |
| Interaction with young intruder | Time with the young intruder | Sex and Genotype | Mixed-effect analysis; **Significant effect of Sex: F(1,34)=8.171, P<0.05; Significant effect of Genotype: F(2, 47)=5.291, P<0.05**; No Sex X Genotype interaction: F(2,47)=0.5835, P=0.573. |
| **Circadian Wheels Test** | | | |
| Tau | Endogenous free-running circadian period | Sex and Genotype | Mixed-effect analysis; No main effect of Sex: F(1,34)=0.1426, P=0.708; No main effect of Genotype: F(2, 44)=1.896, P=0.1622; No Sex X Genotype interaction: F(2, 44)=3.134, P=0.0534. |
| Phase Shift | Change in activity onsets over time | Sex and Genotype | Mixed-effect analysis; No main effect of Sex: F(1,79)=0.9838, P=0.3293; **Significant effect of Genotype: F(2, 79)=5.291, P<0.01;** No Sex X Genotype interaction: F(2, 79)=1.587, P=0.2111. |
| Intradaily Variability in light-dark phase | Measurement of rest-activity rhythm fragmentation | Sex and Genotype | Mixed-effect analysis; No main effect of Sex: F(1,33)=2.280, P=0.1406; **Significant effect of Genotype: F(2, 48)=9.427, P<0.01;** No Sex X Genotype interaction: F(2, 48)=0.05, P=0.9423. |
| Interdaily Stability in light-dark phase | Rest-activity synchronization to the 24-hour light-dark cycle | Sex and Genotype | Mixed-effect analysis; No main effect of Sex: F(1,81)=0.006, P=0.9381; **Significant effect of Genotype: F(2, 81)=4.893, P<0.01;** No Sex X Genotype interaction: F(2, 81)=0.4233, P=0.8583. |
| Bouts per day in light-dark phase | Number of active periods per day | Sex and Genotype | Mixed-effect analysis; No main effect of Sex: F(1,82)=0.7799, P=0.3798; **Significant effect of Genotype: F(2, 82)=11.08, P<0.01;** No Sex X Genotype interaction: F(2, 82)=0.09785, P=0.9069. |
| Bout Length in light-dark phase | Length of time of the active period | Sex and Genotype | Mixed-effect analysis; No main effect of Sex: F(1,82)=2.724, P=0.1027; **Significant effect of Genotype: F(2, 82)=9.85, P<0.01;** No Sex X Genotype interaction: F(2, 82)=0.4743, P=0.624. |
| Revolutions per bout in light-dark phase | Running wheel revolution per active period | Sex and Genotype | Mixed-effect analysis; No main effect of Sex: F(1,82)=3.48, P=0.0657**;** **Significant effect of Genotype: F(2, 82)=18.43, P<0.01;** No Sex X Genotype interaction: F(2, 82)=1.584, P=0.2156. |
