# Supplementary material for: Allelic contribution of Nrxn1α to autism-relevant behavioral phenotypes in mice
Source: PLoS Genet. 2023 Feb 27;19(2):e1010659. doi: 10.1371/journal.pgen.1010659 (PMC9997995; doi:10.1371/journal.pgen.1010659)
Supplement: S2 Table — (PDF) [file pgen.1010659.s013.pdf]

Supplementary Table S2 – Statistical analyses of behavioral phenotypes in the context of sex and genotypes for *Nrxn1* Exon1 deletion mouse model (+/+, ∆Exon1/+)

Behavioral Test	Parameter	Comparison	Results
**3-Box Social interaction Test**			
Preference Test for Social interaction	Time with social cylinder	Sex and Genotype	Mixed-effect analysis; No main effect of Sex: F (1, 53) = 2.706, P=0.1059; No main effect of Genotype: F(1,53)= 0.0095, P=0.9227; No Sex x Genotype interaction: F(1,53) = 0.1454, P=0.7045.
Preference Test for Novel Animal	Time with novel adult mouse	Sex and Genotype	Mixed-effect analysis; No main effect of Sex: F(1,30) =2.098, P=0.1578; **Significant effect of Genotype: F(1,23)=8.9, P<0.01**; No Sex X Genotype interaction: F(1,23)=0.78, P=0.3852.
**Resident-intruder Test**			
Interaction with young intruder	Time with the young intruder	Sex and Genotype	Mixed-effect analysis; No main effect of Sex: F(1,30) =2.098, P=0.1578; **Significant effect of Genotype: F(1,22)=5.648, P<0.05; Significant Sex X Genotype interaction: F(1,22)=5.228, P<0.05.**
**Circadian Wheels Test**			
Tau	Endogenous free-running circadian period	Sex and Genotype	Mixed-effect analysis; No main effect of Sex: F(1,27)=0.04523, P=0.8332; **Significant effect of Genotype: F(1, 22)=10.93, P<0.01; Significant Sex X Genotype interaction: F(1, 22)=4.738, P<0.05.**
Phase Shift	Change in activity onsets over time	Sex and Genotype	Mixed-effect analysis; No main effect of Sex: F(1,24)=0.3, P=0.5889; **Significant effect of Genotype: F(1, 18)=4.697, P<0.05;** No Sex X Genotype interaction: F(1, 18)=0.7945, P=0.3845.
Intradaily Variability in light-dark phase	Measurement of rest-activity rhythm fragmentation	Sex and Genotype	Mixed-effect analysis; No main effect of Sex: F(1, 48)=2.861, P=0.0972; No main effect of Genotype: F(1, 48)=0.04363, P=0.8354; No Sex X Genotype interaction: F(1, 48)=0.2393, P=0.627.
Interdaily Stability in light-dark phase	Rest-activity synchronization to the 24-hour light-dark cycle	Sex and Genotype	Mixed-effect analysis; **Significant effect of Sex: F(1,48)=4.676**, P<0.05; No main effect of Genotype: F(1, 48)=0.498, P=0.4848; No Sex X Genotype interaction: F(1, 48)=0.07704, P=0.7825.
Bouts per day in light-dark phase	Number of active periods per day	Sex and Genotype	Mixed-effect analysis; **Significant effect of Sex: F(1,45)=6.018,** P<0.05; No main effect of Genotype: F(1, 45)=0.0215, P=0.8842; No Sex X Genotype interaction: F(1, 45)=3.359, P=0.0734.
Bout Length in light-dark phase	Length of time of the active period	Sex and Genotype	Mixed-effect analysis; **Significant effect of Sex: F(1,45)=10.18, P<0.01;** No main effect of Genotype: F(1, 45)=0.002318, P=0.9618; No Sex X Genotype interaction: F(1, 45)=1.156, P=0.2881.
Revolutions per bout in light-dark phase	Running wheel revolution per active period	Sex and Genotype	Mixed-effect analysis; **Significant effect of Sex: F(1,45)=13.26, P<0.01;** No main effect of Genotype: F(1, 45)=0.3408, P=0.5572; No Sex X Genotype interaction: F(1, 45)=1.447, P=0.2353.
