# Supplementary material for: Allelic contribution of Nrxn1α to autism-relevant behavioral phenotypes in mice
Source: PLoS Genet. 2023 Feb 27;19(2):e1010659. doi: 10.1371/journal.pgen.1010659 (PMC9997995; doi:10.1371/journal.pgen.1010659)
Supplement: S3 Table — (PDF) [file pgen.1010659.s014.pdf]

Supplementary Table S3 – Statistical analyses of behavioral phenotypes in the context of sex and genotypes for *Nrxn1* Intron17 deletion mouse model (+/+, ΔIntron17/+, ΔIntron17/ΔIntron17)

Behavioral Test	Parameter	Comparison	Results
**3-Box Social interaction Test**			
Preference Test for Social interaction	Time with social cylinder	Sex and Genotype	Mixed-effect analysis; No main effect of Sex: F (1, 28) = 3.394, P=0.0780; No main effect of Genotype: F(2, 46)=0.60, P=0.5523. No Sex X Genotype interaction: F(2, 46)=0.38, P=0.6794.
Preference Test for Novel Animal	Time with novel adult mouse	Sex and Genotype	Mixed-effect analysis; No main effect of Sex: F(1,28) =1.40, P=0.2461; No main effect of Genotype: F(2, 46)=0.4136, P=0.6629; No Sex X Genotype interaction: F(2, 46)=0.73, P=0.4840.
**Resident-intruder Test**			
Interaction with young intruder	Time with the young intruder	Sex and Genotype	Mixed-effect analysis; **Significant effect of Sex: F(1, 69) =22.69, P<0.01;** No main effect of Genotype: F(2, 69)=5.648, P<0.05; **Significant Sex X Genotype interaction: F(2, 69)= 0.3183, P=0.7284.**
**Circadian Wheels Test**			
Tau	Endogenous free-running circadian period	Sex and Genotype	Mixed-effect analysis; No main effect of Sex: F (1, 27) = 2.038, P=0.1649; No main effect of Genotype: F(2, 45)=2.19, P=0.1237. No Sex X Genotype interaction: F(2, 45)=1.596, P=0.2140.
Phase Shift	Change in activity onsets over time	Sex and Genotype	Mixed-effect analysis; No main effect of Sex: F (1, 26) = 2.735, P=0.1102; No main effect of Genotype: F(2, 42)=1.434, P=0.2497. No Sex X Genotype interaction: F(2, 42)=12.717, P=0.0777.
Intradaily Variability in light-dark phase	Measurement of rest-activity rhythm fragmentation	Sex and Genotype	Mixed-effect analysis; **Significant effect of Sex: F(1, 72)=6.588, P<0.05;** No main effect of Genotype: F(2, 72)=1.021, P=0.3653; No Sex X Genotype interaction: F(2, 72)=0.3995, P=0.6722.
Interdaily Stability in light-dark phase	Rest-activity synchronization to the 24-hour light-dark cycle	Sex and Genotype	Mixed-effect analysis; **Significant effect of Sex: F(1, 72)=8.622, P<0.05;** No main effect of Genotype: F(2, 72)=0.8169, P=0.4459; No Sex X Genotype interaction: F(2, 72)=0.4496, P=0.6396.
Bouts per day in light-dark phase	Number of active periods per day	Sex and Genotype	Mixed-effect analysis; No main effect of Sex: F(1,27)=3.856, P=0.0599; No main effect of Genotype: F(2, 46)=0.19415, P=0.8234; No Sex X Genotype interaction: F(2, 46)=0.9034, P=0.4123.
Bout Length in light-dark phase	Length of time of the active period	Sex and Genotype	Mixed-effect analysis; **Significant effect of Sex: F(1,27)=8.265, P<0.01;** No main effect of Genotype: F(2, 46)=0.84738, P=0.4351; No Sex X Genotype interaction: F(2, 46)=1.351, P=0.2691.
Revolutions per bout in light-dark phase	Running wheel revolution per active period	Sex and Genotype	Mixed-effect analysis; **Significant effect of Sex: F(1,27)=9.860, P<0.01;** No main effect of Genotype: F(2, 46)=0.5290, P=0.5927; No Sex X Genotype interaction: F(2, 46)=0.4205, P=0.6592.
